# Comprehensive genomic profiling of breast cancers characterizes germline-somatic mutation interactions mediating therapeutic vulnerabilities

**DOI:** 10.1038/s41421-023-00614-3

**Published:** 2023-12-19

**Authors:** Chao Chen, Cai-Jin Lin, Yu-Chen Pei, Ding Ma, Li Liao, Si-Yuan Li, Lei Fan, Gen-Hong Di, Song-Yang Wu, Xi-Yu Liu, Yun-Jin Wang, Qi Hong, Guo-Liang Zhang, Lin-Lin Xu, Bei-Bei Li, Wei Huang, Jin-Xiu Shi, Yi-Zhou Jiang, Xin Hu, Zhi-Ming Shao

**Affiliations:** 1 Key Laboratory of Breast Cancer in Shanghai, Department of Breast Surgery, Fudan University Shanghai Cancer Center, Shanghai, China; 2 Department of Oncology, Shanghai Medical College, Fudan University, Shanghai, China; 3 Precision Cancer Medical Center Affiliated to Fudan University Shanghai Cancer Center, Shanghai, China; 4 Shanghai-MOST Key Laboratory of Health and Disease Genomics, Shanghai Institute for Biomedical and Pharmaceutical Technologies, Shanghai, China

**Keywords:** Breast cancer, Cancer genomics

## Abstract

Germline-somatic mutation interactions are universal and associated with tumorigenesis, but their role in breast cancer, especially in non-Caucasians, remains poorly characterized. We performed large-scale prospective targeted sequencing of matched tumor-blood samples from 4079 Chinese females, coupled with detailed clinical annotation, to map interactions between germline and somatic alterations. We discovered 368 pathogenic germline variants and identified 5 breast cancer DNA repair-associated genes (BCDGs; *BRCA1/BRCA2/CHEK2/PALB2/TP53*). BCDG mutation carriers, especially those with two-hit inactivation, demonstrated younger onset, higher tumor mutation burden, and greater clinical benefits from platinum drugs, PARP inhibitors, and immune checkpoint inhibitors. Furthermore, we leveraged a multiomics cohort to reveal that clinical benefits derived from two-hit events are associated with increased genome instability and an immune-activated tumor microenvironment. We also established an ethnicity-specific tool to predict BCDG mutation and two-hit status for genetic evaluation and therapeutic decisions. Overall, this study leveraged the large sequencing cohort of Chinese breast cancers, optimizing genomics-guided selection of DNA damaging-targeted therapy and immunotherapy within a broader population.

## Introduction

Cancer is a genetically heterogeneous disease with complex heritable and somatic factors^1^. In the era of precision oncology, integrating genomic profiling into personalized care planning has revolutionized anticancer therapy^2–4^. Previous programs, such as MSK-IMPACT and NCI-MATCH, have convincingly illustrated the importance of molecular profiling via clinical next-generation sequencing^5–10^. However, the focus of translational cancer genomics has primarily been on somatic mutations for the identification of precision medicine targets^11^. Likewise, germline research has mostly involved epidemiological and association-based studies that lack the integration of somatic mutational spectra^12,13^. It is worth highlighting that germline mutations not only contribute to cancer predisposition but also influence responses to both targeted and conventional oncologic therapies^14^. Therefore, it becomes imperative to explore the integration of germline and somatic genomic data to yield further clinical benefits.

Parallel efforts have been made to explore the significance of both germline and somatic aberrations, providing insights into the processes involved in cancer development^1,15^. Recently, studies have begun exploring the interactions between germline and somatic mutations in cancer^16–18^. Notably, research has revealed that early-onset cancers are more often driven by germline mutation load, while late-onset malignancies are more dependent on somatic mutation burden^1,16^. However, most of these studies have focused on Caucasian and African populations, and a direct transfer of these findings to Asian populations would likely be unfeasible due to ethnic specificity^19,20^. Additionally, few studies have addressed whether germline-somatic mutation interactions affect therapeutic sensitivity. As a result, it remains unclear whether common threads that define key mechanisms of germline-somatic variant interactions could be harnessed to improve survival and refine genomics-guided treatment in Asian populations.

These challenges necessitate a thorough investigation into specific germline-somatic mutation interactions in Asian populations. Thus, we initiated the Fudan University Shanghai Cancer Center-Breast Cancer (FUSCC-BC) program, which integrates paired tumor-blood genomic information with clinical records to elucidate the functions of germline-somatic mutation interactions. The categories of germline-somatic mutation interactions mainly include the co-occurrence and mutual exclusivity between germline and somatic mutations of different driver genes, as well as two-hit inactivation of the same driver genes^15,18^. With the significant expansion of our database to 4079 cases, we updated the somatic genomic landscape based on our previous study^21^ and systematically characterized the functional implications of germline-somatic mutation interactions for tumorigenesis, treatment efficacy, and survival outcomes. Furthermore, leveraging our multiomics triple-negative BC (TNBC) cohort, we revealed the clinical and biological implications of two-hit events. Finally, we developed a mutation prediction algorithm to facilitate its broader application in the clinical setting.

## Results

### Characteristics of study patients and prospective targeted sequencing samples in the FUSCC-BC cohort

**Fig. 1:**
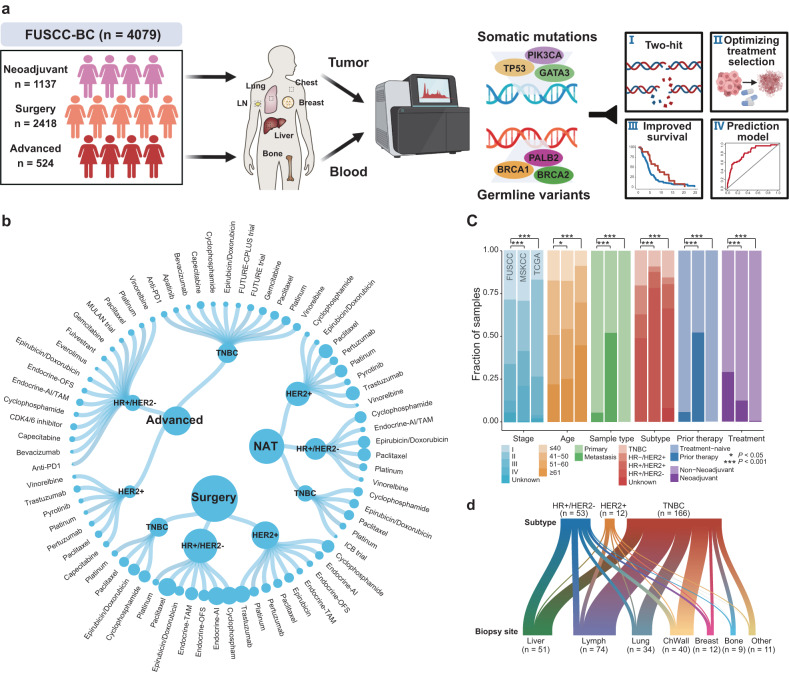
Schematic workflow and clinical features of study patients and prospective sequencing samples in the FUSCC-BC cohort. **a** Schematic representation of workflow for the targeted sequencing analyses in our study, including cohort establishment, sample preparation, panel sequencing, and optimization of genomics-guided treatment. **b** Treatment information for each molecular subtype in the surgery cohort, neoadjuvant cohort, and advanced cohort. The size of the circles represents the number of patients with the corresponding drugs. NAT, neoadjuvant therapy. **c** The clinical characteristics of our cohort compared with those reported in prior breast cancer sequencing studies (MSKCC and TCGA). See also Supplementary Table S2. **d** The distribution of biopsied metastatic sites grouped by molecular subtype. The width of connecting lines is proportional to the number of patients with the respective molecular subtype and biopsy site. A portion of **a** was created with BioRender.com.

We conducted prospective clinical sequencing of samples from 4079 breast cancer patients treated at FUSCC between April 2018 and June 2021 (Fig. 1a). Matched tumor and blood samples were subjected to in-depth sequencing via our established targeted sequencing and analysis platform to capture potential germline and somatic aberrations. The patients were manually subdivided into three cohorts: 2418 early-stage patients who subsequently received surgery (surgery cohort), 1137 locally advanced breast cancer patients who received neoadjuvant therapy (neoadjuvant cohort), and 524 metastatic patients who received salvage therapy (advanced cohort). The targeted sequencing cohort included numerous clinical trial cohorts involving > 30 different drugs (Fig. 1b). Participants were enrolled in specific clinical trials based on their individual genomic and histopathologic characteristics. For example, clinical trials of neoadjuvant therapy (NCT04215003), the FUTURE trial (NCT03805399)^22^, the FUTURE-CPLUS trial (NCT04129996)^23^, and another MULAN trial (NCT04355858) were designed to practice genomics-guided treatment. A detailed annotation of clinicopathologic features, treatment modalities, and longitudinal follow-up is available in Supplementary Table S1. Importantly, all clinical information and sequencing data were constantly updated and uploaded to our Fudan Data Portal (https://data.3steps.cn/cdataportal/study/summary?id=FUSCC_BRCA_panel_4000), an open-access database for human cancer genomics research.

We summarized and compared the clinical features of our cohorts with other databases, including the Memorial Sloan Kettering Cancer Center (MSKCC) and The Cancer Genome Atlas (TCGA) (Fig. 1c and Supplementary Table S2). These comparisons revealed that our cohort exhibited a younger age of onset, a greater proportion of patients with TNBC, and a lower number of metastatic samples (*n* = 231; Fig. 1d).

### Comprehensive somatic genomic landscape of Chinese breast cancer

**Fig. 2:**
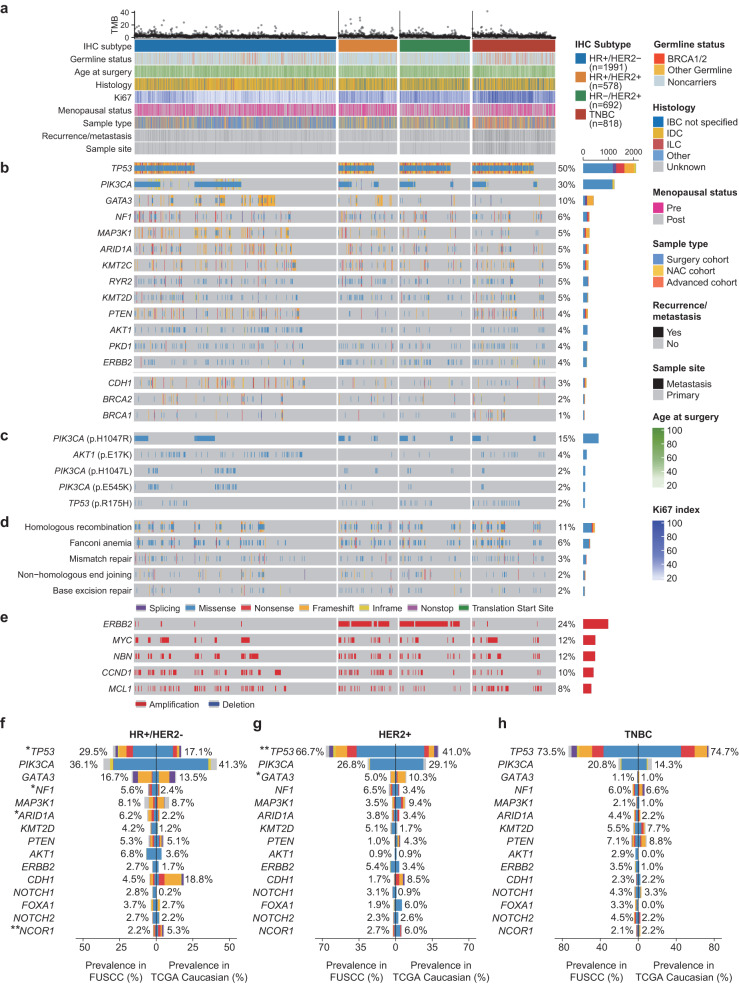
Somatic genomic landscape and ethnicity-specific genomic mutations in prospectively sequenced Chinese breast cancers. **a** A total of 4079 breast cancer samples with clinical data were ordered by molecular subtype and mutation profile, with clinical and molecular features annotated below. **b** The top thirteen somatically altered genes that were mutated in at least 4% of the cases (upper); somatic *CDH1* and *BRCA1/2* mutations (lower). Genes were ordered by the mutation frequencies, and the mutation counts for each gene are provided on the right side. **c** Hotspot mutations (frequency > 2%) in the Chinese population. **d** DDR pathway mutations in the FUSCC-BC cohort. **e** Copy number variations (CNVs) of cancer-related genes categorized by molecular subtype. **f**–**h** Comparison of somatic mutations in the HR^+^/HER2^–^, HER2^+^, and TNBC subtypes between the FUSCC and TCGA Caucasian cohorts. Logistic regression accounts for age and histology. *P < 0.05, **P < 0.01.

Across the FUSCC-BC cohort with targeted sequencing data, we detected a total of 23,844 protein-altering and splice-site variants, including 18,087 missense, 1845 nonsense, 20 nonstop, 11 translation start-site, 3218 insertion/deletion (indel), and 663 splice-site somatic mutations. The most prominent cancer-related mutations in our cohort occurred in *TP53* (49.9%), *PIK3CA* (30.1%), *GATA3* (10.0%), *NF1* (6.0%), and *MAP3K1* (5.4%) (Fig. 2a, b). The most prevalent hotspot mutation in our cohort was *PIK3CA* p.H1047R (14.9%), followed by *AKT1* p.E17K (3.6%), *PIK3CA* p.H1047L (2.1%), *PIK3CA* p.E545K (2.0%), and *TP53* p.R175H (1.7%) (Fig. 2c). Numerous genes and hotspots showed distinct molecular distributions of alterations among different immunohistochemical (IHC) subtypes (Supplementary Fig. S1a–c). For example, *TP53* mutations were highly enriched in TNBC (73.5%), but the lowest proportion was shown in the hormone receptor (HR)^+^/human epidermal growth factor receptor 2 (HER2)^–^ (29.5%) subtype. Likewise, the highest frequency of *BRCA1* mutations was present in TNBC (2.8%) compared with other IHC subtypes. The top five somatic copy number alterations were in *ERBB2* (24.4%), *MYC* (11.8%), *NBN* (11.6%), *CCND1* (10.1%), and *MCL1* (8.0%) (Fig. 2e).

DNA damage repair (DDR) pathway deficiency is a significant determinant of cancer progression and treatment response^24^. Thus, we further established the landscape of DDR pathway mutations (Fig. 2d and Supplementary Fig. S1d), which demonstrated high mutation frequencies in homologous recombination (HR, 10.8%), Fanconi anemia (FA, 6.5%), and mismatch repair (MMR, 3.2%) pathways.

Furthermore, we compared the somatic mutation frequency of FUSCC with that of MSKCC, TCGA Caucasian or African, and GENIE Caucasian or African cohorts within each molecular subtype (Fig. 2f–h and Supplementary Fig. S2). The differences were largely centered on the HR^+^/HER2^–^ subtype. Specifically, in comparison to the TCGA Caucasian and MSKCC cohorts, the FUSCC cohort showed a higher frequency of *NF1* (5.6% in FUSCC vs 2.4% in TCGA Caucasian, *P* = 0.02; 5.6% in FUSCC vs 4.4% in MSKCC, *P* = 0.0009) and *TP53* (29.5% in FUSCC vs 17.1% in TCGA Caucasian, *P* = 0.02; 29.5% in FUSCC vs 28.2% in MSKCC, *P* = 0.01) mutations (Fig. 2f and Supplementary Fig. S2d). In addition, FUSCC exhibited an increased *ARID1A* (6.2% in FUSCC vs 2.2% in TCGA Caucasian, *P* = 0.02) mutation and a decreased *NCOR1* (2.2% in FUSCC vs 5.3% in TCGA Caucasian, *P* = 0.007) mutation compared with TCGA Caucasians. Compared to HER2^+^ breast cancer in the TCGA Caucasian cohort, we found *TP53* (66.7% in FUSCC vs 41.0% in TCGA Caucasian, *P* = 0.001) with a higher frequency and *GATA3* (5.0% in FUSCC vs 10.3% in TCGA Caucasian, *P* = 0.01) with a lower frequency in our cohort (Fig. 2g). Similar results were detected in the comparison with the GENIE Caucasian cohort (Supplementary Fig. S2g). However, no difference was observed in the comparison with TNBC in the TCGA Caucasian cohort (Fig. 2h). Similarly, when compared to the TCGA African cohort, no significant disparities were found (Supplementary Fig. S2a–c). The lack of significant differences might be attributed to the relatively small size of the TCGA African cohort. Moreover, when compared with the GENIE African cohort, we detected an increased *PIK3CA* (30.1% in FUSCC vs 25.9% in GENIE African, *P* = 0.003) mutation and a decreased *GATA3* (10.0% in FUSCC vs 13.7% in GENIE African, *P* = 0.0008) mutation in the FUSCC cohort (Supplementary Fig. S2h). Overall, we revealed the landscape of somatic mutations and discerned the distinctions among populations of Asian, Caucasian, and African ancestries.

### Comprehensive germline profiling in Chinese breast cancer

**Fig. 3:**
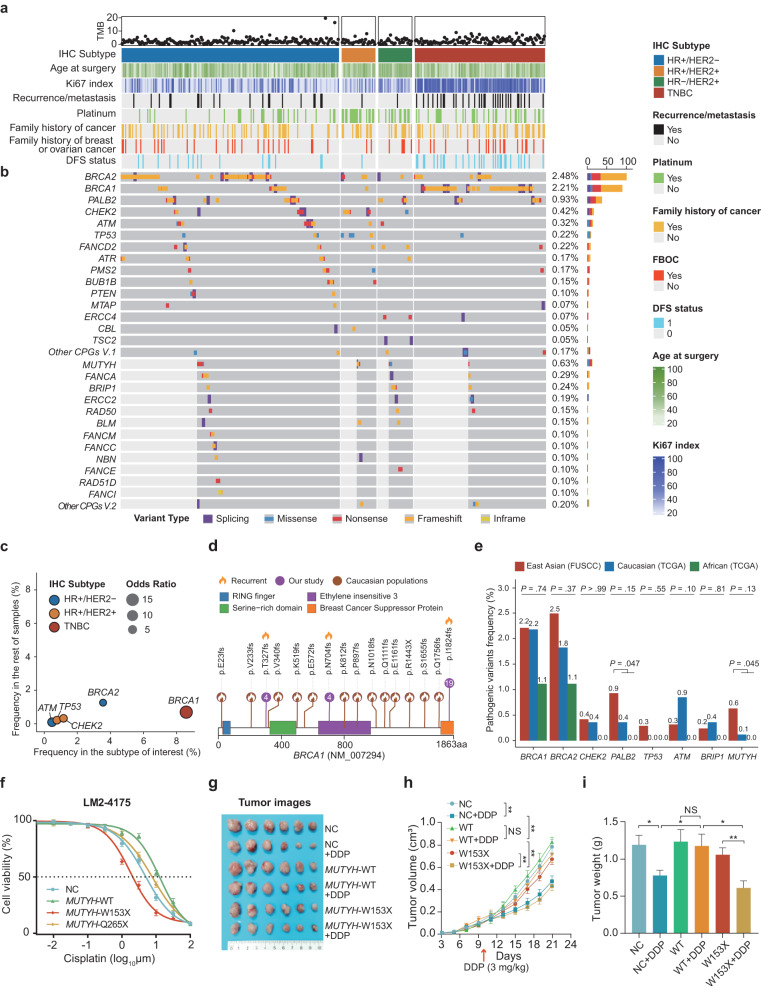
Pathogenic germline mutational profile of Chinese breast cancer patients. **a,** **b** The spectrum of pathogenic germline variants in the FUSCC-BC cohort. Each column represents one patient. The upper part (**a**) shows the patient’s clinical characteristics: tumor mutation burden (TMB), molecular subtype, age at surgery, Ki67 index, recurrent or metastatic status, platinum treatment, family history of cancer, FBOC, and DFS status. The lower part (**b**) shows the spectrum of pathogenic germline variants. Other cancer predisposition genes (CPGs) version 1 (Other CPGs V.1) represents one pathogenic variant in each of the *APC*, *FH*, *MLH1*, *RET*, *RUNX1*, *SMARCA4*, and *VHL* genes. Other CPGs V.2 represents one pathogenic variant in each of the *BARD1*, *FANCL, MSH6*, and *RAD54L* genes. White bar plots indicate that these samples were not tested for the corresponding row of genes. **c** Significant enrichment of germline variants in different molecular subtypes. The *x*-axis is the frequency of the gene in the subtype of interest, and the corresponding *y*-axis is the frequency of the gene in the other subtypes. The size of the dot represents the odds ratio (OR). Statistical analysis was carried out using Fisher’s exact test. **d** Lolliplot of high-frequency *BRCA1* pathogenic variant distribution in Caucasian populations and our study. Caucasian populations included Ashkenazi Jewish, Norwegian, Swedish, French, Dutch, and Italian populations. **e** Comparison of germline alterations between the FUSCC-BC, TCGA Caucasian, and TCGA African cohorts. Statistical analysis was carried out using Fisher’s exact test. **f** Cell viability following treatment with cisplatin at the indicated concentration measured in LM2-4175 cells stably expressing empty vector, *MUTYH* WT, *MUTYH* W153X, and *MUTYH* Q265X. **g**–**i** In vivo growth of breast cancer cells (LM2-4175) stably expressing empty vector, *MUTYH* WT or *MUTYH* W153X. Six mice per group. After inoculation for 10 days, the mice were administered with cisplatin (DDP) (3 mg/kg) by intraperitoneal injection every 2 days for 6 times. The representative tumor image, volume quantification and tumor weight results are shown. NC means negative control. Statistical analysis was performed using a two-tailed unpaired Student’s *t-*test for the in vivo experiments. Error bars represent means ± SD (*n* = 6 per group). *P < 0.05, **P < 0.01, ***P < 0.001, and ns, not significant.

In our FUSCC-BC cohort, we had blood DNA samples available for the 4079 patients. By sequencing cancer susceptibility genes, we identified 28,507 variants with uncertain significance and 368 pathogenic or likely pathogenic (P/LP) germline variants (Supplementary Fig. S3a). Among the 368 P/LP variants, 96 (26.1%) were novel and had not been reported in the dbSNP and ClinVar databases (Supplementary Table S3). The 368 germline variants consisted of 340 truncating and 28 missense mutations (Supplementary Fig. S3b). In our study, 350 (8.6%) patients carried at least one P/LP variant (Supplementary Fig. S3c), and among them, 18 patients (0.4%) had two pathogenic variants (Supplementary Table S3). The most prevalent P/LP mutations were in *BRCA2* (2.5%), followed by *BRCA1* (2.2%), *PALB2* (0.9%), *MUTYH* (0.6%), *CHEK2* (0.4%), *ATM* (0.3%), and *TP53* (0.2%) (Fig. 3a, b).

We further summarized the molecular distribution of P/LP alterations in different IHC subtypes (Fig. 3c). In the HR^+^/HER2^–^ subtype, the frequency of *BRCA2* and *ATM* variants was significantly higher than that in the other subtypes. *CHEK2* and *TP53* mutations were more common in HR^+^/HER2^+^ patients, while *BRCA1* mutations were more prevalent in TNBC patients. Furthermore, we explored the clinicopathological features of high-frequency germline mutation carriers after adjusting for molecular subtype and histology (Supplementary Table S4). Germline *BRCA1/2* carriers were associated with a younger age at diagnosis, greater Ki67 index values, higher frequencies of bilateral breast cancer, positive lymph node status, and a family history of breast or ovarian cancer (FBOC). In addition, pathogenic *BRCA1* variants, but not *BRCA2* variants, correlated with higher histological grades. Pathogenic *PALB2* variants were also related to a higher Ki67 proliferative fraction and an earlier onset age. Of note, germline *MUTYH* variants were associated with metastatic status. Thus, we sought to determine whether these pathogenic variants correlated with aggressive and metastatic phenotypes. Carriers with *BRCA1* variants exhibited worse disease-free survival (DFS) outcomes (*P* = 0.002; Supplementary Fig. S3d). However, in the multivariate analysis, adjusting for age, FBOC, nuclear grade, tumor size, lymph node status, ER, PR, HER2 status, and treatment, this association was not significant (*P* = 0.085; Supplementary Fig. S3e).

Notably, among the 4079 patients, three recurrent *BRCA1* variants had a relatively high frequency (≥ 4 cases), namely, p.T327fs (4 cases; 0.98‰), p.N704fs (4 cases; 0.98‰) and p.I1824fs (19 cases; 4.66‰), accounting for 30% of the *BRCA1* mutations (Fig. 3d). Interestingly, these recurrent *BRCA1* variants in the Chinese population differed significantly from those found in Caucasian populations. Within our cohort, the two most common *PALB2* variants were p.M723fs and p.X1038_splice, each with 4 cases (Supplementary Fig. S5c), which also differed from the Caucasian population. To further investigate germline diversity among different ethnicities, we analyzed additional mutational spectrum data from TCGA^18^. Our analysis showed that the frequency of germline mutations was almost equivalent among East Asian, Caucasian, and African populations (Fig. 3e). Interestingly, the prevalence of *MUTYH* and *PALB2* was higher in East Asians than in non-East Asians. Germline *PALB2* and *BRCA1/2* mutations have been identified as therapeutic targets associated with sensitivity to poly(ADP-ribose) polymerase (PARP) inhibitors (PARPi) and platinum agents^25,26^. However, studies exploring the drug sensitivity of *MUTYH* variants are lacking. In this study, we generated stable cell lines overexpressing negative control (NC), wild-type (WT), or mutant *MUTYH* (W153X and Q265X) (Supplementary Fig. S4a–d). Three different cell lines (H578T, LM2-4175, and T47D) with *MUTYH* variants, especially recurrent mutations involving W153X, were sensitive to cisplatin (Fig. 3f and Supplementary Fig. S4e, f). This sensitivity was further confirmed by in vivo experiments (Fig. 3g–i) and colony formation assays (Supplementary Fig. S4g–i). Moreover, exogenous *MUTYH*-WT showed platinum resistance compared to the negative control. Collectively, our study provided a comprehensive overview of germline mutations, identified racial disparities, and validated the platinum sensitivity of recurrent *MUTYH* variants.

### Clinical characteristics of germline alterations and comparison with the general population

**Fig. 4:**
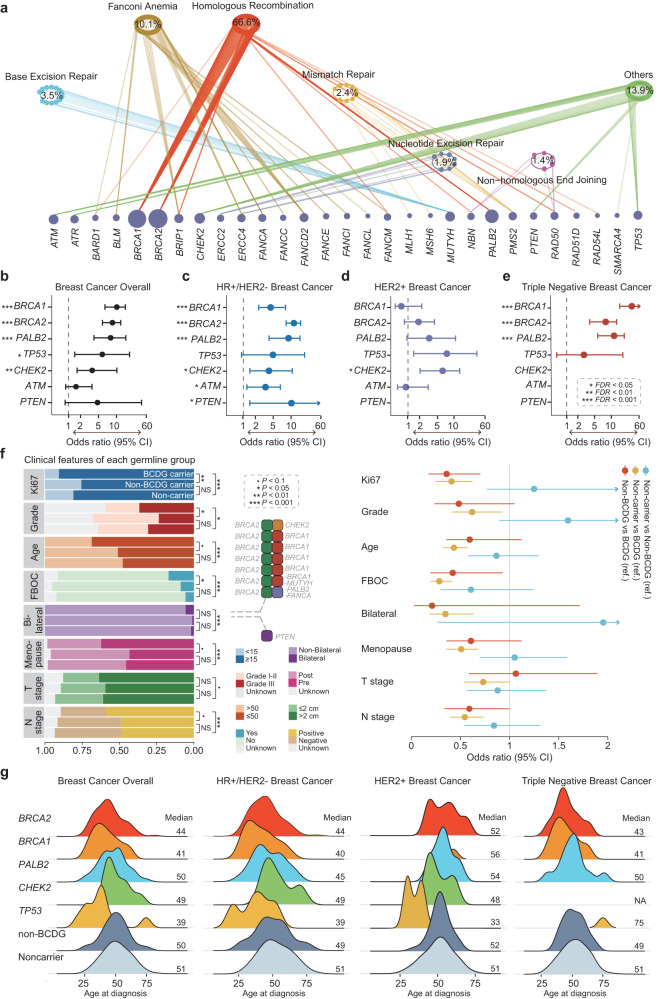
Clinical characteristics of germline alterations and comparison with the general population. **a** The prevalence of pathogenic germline variants in the DDR pathway. **b**–**e** Overall breast cancer risk and molecular subtypes associated with pathogenic germline variants in a population-based study. ORs and 95% CIs are shown for overall breast cancer, HR^+^/HER2^–^ breast cancer, HER2^+^ breast cancer, and TNBC associated with germline variants. The control cohort was obtained from the East Asian populations (*n* = 9977) in GnomAD. The case‒control association was assessed by Fisher’s exact test, adjusted by the false discovery rate (FDR). One asterisk indicates FDR < 0.05, two indicate FDR < 0.01, three indicate FDR < 0.001, and no asterisk on the left of the gene name indicates no significance. The detailed *P* values are available in Supplementary Tables S6 and S7. **f** Comparisons of clinical features in each germline mutation group categorized by BCDG mutations; the panel on the upper right details the germline variants with a history of contralateral breast cancer. Logistic regression accounts for clinical subtypes and histology. A forest plot presents the adjusted OR and 95% CI of the comparison of clinical features among different germline groups. **g** Age at diagnosis was compared according to each germline mutation group among different molecular subtypes. A smaller mountain indicates one patient, such as a *BRCA1* carrier in HER2^+^ breast cancer and a *TP53* carrier in TNBC. The *P* values were calculated by the Wilcoxon signed-rank test. •*P* < 0.1, *P < 0.05, **P < 0.01, ***P < 0.001, and ns, not significant.

Germline mutations in DDR genes play an important role in cancer risk and treatment response^27^. Therefore, we curated a list of DDR genes (Supplementary Table S5) and observed that most germline variants (95%) occurred in these genes. Then, we analyzed the DDR pathways with germline variants and revealed fairly high mutation rates in the HR (67%) and FA (10%) pathways (Fig. 4a).

Breast cancer predisposition genes not only influence breast cancer risk but also impact therapeutic response^28^. The increasing number of public genome databases of cancer-free populations has facilitated the discovery of breast cancer predisposition genes^27,29^. To evaluate breast cancer predisposition genes in the Chinese population, we performed a population-based case‒control study, identifying five genes significantly correlated with breast cancer risk (Fig. 4b). These genes included *BRCA2* (odds ratio (OR), 9.02; 95% CI, 5.87–14.27), *BRCA1* (OR, 10.69; 95% CI, 6.58–18.14), *PALB2* (OR, 8.52; 95% CI, 4.26–18.5), *CHEK2* (OR, 4.17; 95% CI, 1.8–10.2), and *TP53* (OR, 5.51; 95% CI, 1.54–24.51) (Supplementary Table S6). Therefore, these five genes were identified as breast cancer predisposition genes in the Chinese population. The details of the alterations in these five genes are shown in Supplementary Fig. S5. Furthermore, we analyzed the breast cancer predisposition genes in each molecular subtype (Supplementary Fig. S4c–e), resulting in the characterization of *ATM* (OR, 2.91; 95% CI, 1.25–6.45) and *PTEN* (OR, 10.04; 95% CI, 1.44–111.08) as predisposition genes exclusively in the HR^+^/HER2^–^ subtype (Supplementary Table S7).

We categorized the patients into three categories: (1) mutations in five BCDGs (*BRCA1/BRCA2/CHEK2/PALB2/TP53*) identified in our study; (2) mutations in non-BCDGs (33 genes); and (3) no mutations. BCDG mutations were correlated with a younger age of onset, higher Ki67 index values, increased frequencies of high histological grades, positive lymph node status, bilateral breast cancer, and FBOC (Fig. 4f and Supplementary Table S8). We discovered that *BRCA1*, *BRCA2*, *PALB2*, and *TP53* carriers exhibited a younger age of onset among the overall population, whereas *CHEK2* carriers did not demonstrate this association (Fig. 4g). Specifically, in HR^+^/HER2^–^ breast cancers, *BRCA1*, *BRCA2*, and *TP53* carriers had an earlier age of onset than non-BCDG carriers and noncarriers. In contrast, *PALB2* carriers were only slightly younger than noncarriers (*P* = 0.047) but were not significantly different from non-BCDG carriers (*P* = 0.355). Interestingly, only *TP53* carriers had an earlier age at diagnosis in HER2^+^ breast cancers. As anticipated, carriers with *BRCA1* and *BRCA2* variants also had an earlier age of onset in TNBC. In the multivariate logistic regression analysis, age at diagnosis (OR, 0.95; 95% CI, 0.94–0.97; *P* < 0.001), Ki67 index (OR, 1.01; 95% CI, 1.00–1.02; *P* = 0.009), TNBC (OR, 2.88; 95% CI 1.88–4.39; *P* < 0.001), history of contralateral breast cancer (OR, 3.01; 95% CI, 1.04–7.57; *P* = 0.03), and family history of cancer (OR, 1.53; 95% CI, 1.05–2.21; *P* = 0.025) were the most useful predictors of germline mutations in BCDG (Supplementary Table S9). Overall, we identified five BCDGs and characterized the clinical features of these BCDG carriers.

### Germline and somatic mutation interactions in the molecular biology of breast cancer

**Fig. 5:**
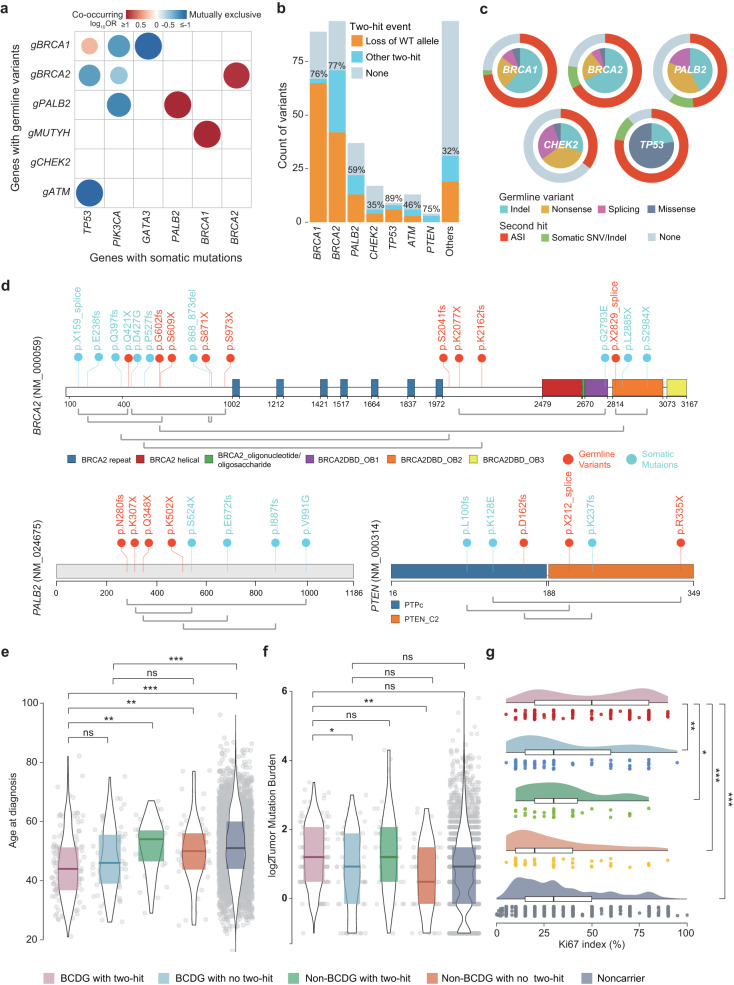
Identification and phenotypic consequences of two-hit events in Chinese breast cancers. **a** Significant co-occurrence (red) and mutual exclusivity (blue) between germline variants and somatic mutations. Spectrum bar: log_10_ (OR); different shades of color indicate the scale of the value. **b** Counts of germline variants showing the various types of two-hit events in cancer predisposition genes. **c** Percentages and types of pathogenic germline variants (inner ring) and second hits (outer circle) in the BCDG variants, including *BRCA1*, *BRCA2*, *PALB2*, *CHEK2*, and *TP53* variants. **d** Biallelic events of pathogenic variants coupled with somatic mutations in the same gene in *BRCA2*, *PALB2*, and *PTEN*. Germline variants are colored red, and somatic mutations are colored blue. Germline and somatic mutations determined in the same patient are connected by light gray lines. **e**–**g** Age at diagnosis, TMB, and Ki67 index comparisons among the groups of BCDG mutation carriers with two-hit inactivation, BCDG carriers without two-hit inactivation, other germline carriers with two-hit inactivation, other germline carriers without two-hit inactivation, and noncarriers. Logistic regression accounts for clinical subtypes, histology, and sample sites. *P < 0.05, **P < 0.01, ***P < 0.001, and ns not significant.

To elucidate the mechanisms underlying germline-somatic mutation interactions in breast cancer initiation and progression, we curated and functionally annotated cancer driver genes (Supplementary Fig. S6a) and detected germline-somatic variant interactions occurring in the same or distinct driver genes. First, we observed that specific somatic mutations were significantly influenced by germline variations in carriers (Supplementary Fig. S6b, c). For example, germline carriers had a higher frequency of *BRCA2* somatic mutations but a lower frequency of *PIK3CA*/*GATA3* somatic mutations than noncarriers. Similarly, BCDG carriers displayed decreased *PIK3CA* and *GATA3* mutations. These results suggested potential co-occurrence and mutual exclusivity. Therefore, we further investigated the co-occurrence and mutual exclusivity between germline and somatic mutations (Fig. 5a). We confirmed the previously reported mutual exclusivity between germline *BRCA1*/*BRCA2* variants (g*BRCA1/2*) and *PIK3CA* somatic mutations as well as the co-occurrence of g*BRCA1* and *TP53* somatic mutations^30–32^. Moreover, *ATM* germline mutations were mutually exclusive with *TP53* somatic mutations, indicating potential functional redundancy between *TP53* and *ATM* inactivation^33^. Interestingly, we observed mutual exclusivity between g*PALB2* and *PIK3CA* somatic mutations, and the co-occurrence of *MUTYH* germline mutations with *BRCA1* somatic mutations. In addition, we discovered co-occurring events in the same genes, including *BRCA2* and *PALB2*, representing two-hit events.

Germline-somatic mutation interactions are best exemplified by Knudson’s seminal “two-hit hypothesis”^34^, which suggests that a tumor will develop after an initial germline inactivation in one allele coupled with the somatic mutation of the other. To better understand the biological functions of the pathogenic variants, we evaluated 215 two-hit events in the FUSCC-BC cohort. These events were more prevalent in BCDGs (72%), specifically *BRCA1* (76%), *BRCA2* (77%), *PALB2* (59%), *CHEK2* (35%), and *TP53* (89%), than in non-BCDGs (35%; Fig. 5b, c). This discrepancy may be attributed to the composition of carriers, with 253 BCDG carriers consisting of 236 high-penetrance and 17 moderate-penetrance carriers, while the 97 non-BCDG carriers primarily comprised 19 high-penetrance, 20 moderate-penetrance, and 58 low/recessive/uncertain-penetrance carriers. In brief, the enrichment of two-hit inactivation of high-penetrance genes underscores their significance as important drivers of tumorigenesis.

Most of the two-hit events exhibited a pronounced allele-specific imbalance (ASI) (Fig. 5c). Another type of two-hit inactivation involves a P/LP germline variant followed by a somatic mutation in the other allele of the same gene. We identified 21 biallelic events (Fig. 5d). Nine germline variants of *BRCA2*, such as p.Q421X, p.G602fs and p.Q609X, were accompanied by somatic *BRCA2* mutations. Four cases harboring different germline truncations, including p.N280fs, p.K502X, p.K307X and p.Q348X, carried *PALB2* somatic mutations. Similarly, three cases carrying three *PTEN* germline truncations (p.D162fs, p.X212_splice and p.R335X) were also coupled with *PTEN* somatic alterations.

We performed exploratory analyses to further observe differences in clinical features between ASI and biallelic mutation carriers (Supplementary Fig. S6d). We observed that biallelic mutations occurred more frequently in the HR^+^ group than ASI. Next, we attempted to identify clinical and molecular features of germline pathogenic alleles and their related somatic alterations in terms of zygosity. Our findings revealed a significant correlation between BCDG carriers, especially those with two-hit inactivation, a younger onset age, higher tumor mutation burden (TMB), and elevated Ki67 index compared to other germline carriers and noncarriers (Fig. 5e–g). Overall, we characterized the pattern of germline-somatic mutation interactions in Chinese breast cancer patients. We found that these interactions predominantly involved two-hit events, suggesting a potential mechanism of tumorigenesis.

### Therapeutic impact and biological features of two-hit inactivation

**Fig. 6:**
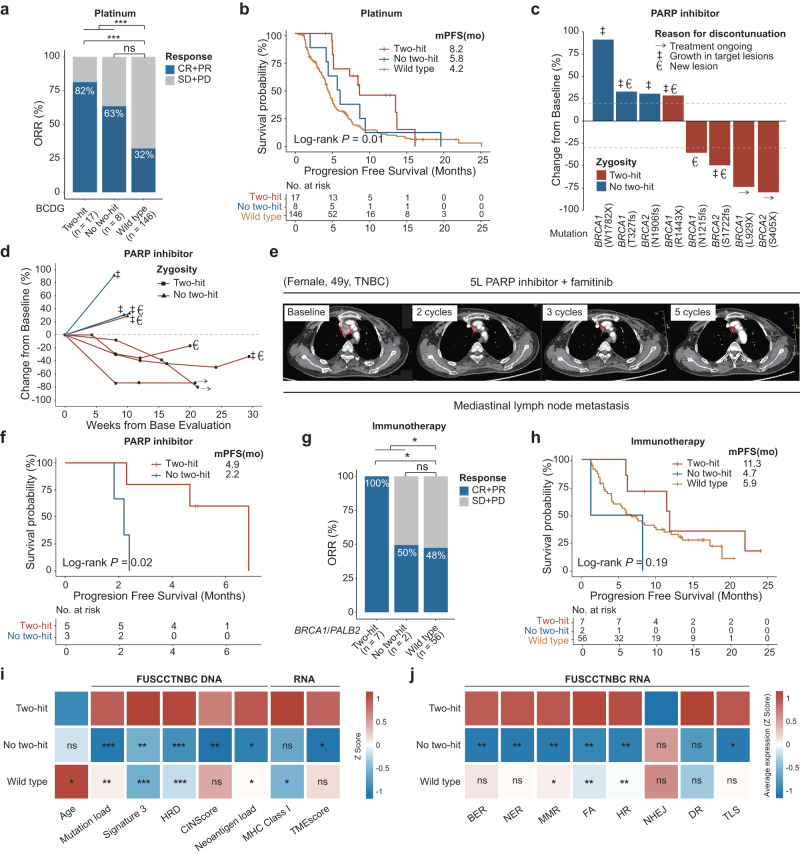
Therapeutic analyses of BCDG mutation and two-hit inactivation. **a** ORR grouped by BCDG germline mutation status in the platinum-treated advanced cohort of HER2^−^ patients. *P* values were determined by Fisher’s exact test. **b** Kaplan–Meier curves of PFS by zygosity category in the platinum-treated advanced cohort of HER2^−^ patients. **c** The best percentage change from baseline in the sum of the longest diameters of target lesions in clinical trials of PARPi, as assessed by the investigators (*n* = 8); zygosity is shown by color coding of bars; the best overall response includes assessment of target, nontarget, and new lesions via RECIST v1.1. **d** Longitudinal change from baseline in the sum of the longest diameters of target lesions in clinical trials of PARPi. **e** Clinical course of fifth-line (5 L) PARPi and famitinib treatment in a patient with mediastinal lymph node metastasis harboring a *BRCA1* L929X mutation. Computed tomography (CT) images are shown for baseline, 2 cycles, 3 cycles, and 5 cycles post treatment. Red circles indicate measurable lesions. **f** Kaplan–Meier curves of PFS by zygosity category in clinical trials of PARPi. **g** ORR grouped by *BRCA1*/*PALB2* germline mutation status in the ICI-treated advanced cohort of HER2^−^ patients. *P* values were calculated using Fisher’s exact test. **h** Kaplan–Meier curves of PFS by two-hit inactivation status in the ICI-treated advanced cohort of HER2^−^ patients. **i** Graphical summary of clinicogenomic and transcriptomic signatures in two-hit tumors. All pairwise comparisons are with the two-hit group. **j** Association between DNA damage response score and two-hit status. All pairwise comparisons are with the two-hit group. CR complete response, PR partial response, SD stable disease, PD progressive disease. *P < 0.05, **P < 0.01, ***P < 0.001, and ns not significant.

To further explore the therapeutic impact of BCDG mutations and two-hit inactivation on clinical outcomes, we investigated the response per BCDG group in several treatment cohorts and prospective clinical trials. In the platinum-treated advanced HER2^−^ cohort, BCDG carriers, particularly those with two-hit inactivation (*P* = 7.01 × 10^−5^), showed a higher objective response rate (ORR; *P* = 3.74 × 10^−5^) and longer median progression-free survival (PFS) than WT patients (two-hit vs no two-hit vs WT: 8.2 vs 5.8 vs 4.2 months, *P* = 0.01; Fig. 6a, b). Moreover, patients harboring mutations in non-*BRCA1/2* BCDGs, including *PALB2*, *CHEK2*, and *TP53*, exhibited a trend toward improved median PFS compared to noncarriers (8.9 months vs 4.2 months; Supplementary Fig. S7a).

The FUTURE trial (NCT03805399)^22^ was a phase Ib/II, open-label, multicenter umbrella study of patients with refractory metastatic TNBC. In this trial, patients of the basal-like immune suppressed subtype with a g*BRCA* variant were administered with a PARPi. Similarly, g*BRCA*-positive patients participating in the MULAN trial (NCT04355858), a prospective, single-center, open-label, umbrella-shaped phase II clinical study for patients with HR^+^/HER2^–^ endocrine-resistant advanced breast cancer, also received PARPi. To validate the role of two-hit inactivation in the efficacy of PARPi, we analyzed the outcomes of all assessable metastatic patients treated with PARPi from the FUTURE and MULAN trial cohorts. Among these patients, four (80%) out of the five two-hit carriers achieved an objective response at their first postbaseline evaluation, whereas three monoallelic cases experienced progressive disease (Fig. 6c, d). Remarkably, a TNBC patient with mediastinal lymph node metastasis who had a two-hit inactivation of a germline *BRCA1* mutation exhibited a complete response following fifth-line treatment with PARPi and famitinib (Fig. 6e). Furthermore, biallelic *BRCA1/2* mutation carriers treated with PARPi had a better PFS benefit than monoallelic carriers (4.9 months vs 2.2 months, *P* = 0.02; Fig. 6f).

Germline alterations in the DDR pathway may be reliable biomarkers of immunotherapy response^35^. Based on the FUTURE and FUTURE-CPLUS trials, we further evaluated the association between two-hit status and the response to immune checkpoint inhibitors (ICIs). Our study found that advanced HER2^−^ patients with *BRCA1/PALB2* mutations, especially two-hit carriers, had a higher ORR when treated with ICIs (*P* = 0.03; Fig. 6g). Compared with monoallelic carriers and noncarriers, two-hit carriers showed a trend toward a longer median PFS (two-hit vs no two-hit vs WT: 11.3 vs 4.7 vs 5.9 months, *P* = 0.19; Fig. 6h).

To gain insights into the mechanisms underlying the increased drug sensitivity resulting from two-hit events, we leveraged the TNBC multiomics cohort to investigate the biological properties of two-hit inactivation. We first observed a correlation between double-hit events in DNA repair genes and a younger age at diagnosis, higher mutation load, increased contribution of mutational signature 3, and elevated scores for homologous recombination deficiency (HRD) and chromosome instability (CIN) (Fig. 6i). These findings demonstrated the presence of increased genomic instability in two-hit tumors. Additionally, two-hit tumors exhibited higher scores for six DDR pathways, including the MMR, FA, and HR pathways (Fig. 6j). The results were further supported by Gene Set Enrichment Analysis (GSEA), which confirmed the upregulation of DNA double-strand break repair signaling (Supplementary Fig. S7b). Notably, the two-hit group exhibited an elevated neoantigen load and a higher MHC class I signature score (Fig. 6i). In-depth analysis of MHC class I at the molecular level revealed upregulation of *HLA-A*, *TAP1*, and *TAP2* (Supplementary Fig. S7c). Moreover, we observed a higher score for the tumor microenvironment (TME) signature in the two-hit group (Fig. 6i), which may potentially explain the favorable responses of two-hit tumors to immunotherapies. CIBERSORT analysis further revealed increased infiltration of T follicular helper cells, M1 macrophages, memory B cells, and activated natural killer (NK) cells (Supplementary Fig. S7d).

Collectively, two-hit carriers demonstrated improved clinical outcomes in response to platinum-based therapies, PARPi, and ICIs. These favorable responses could be attributed to two key factors: increased genomic instability and the immune-activated TME.

### Predicting the likelihood of carrying BCDG mutations and two-hit events in the Chinese population

**Fig. 7:**
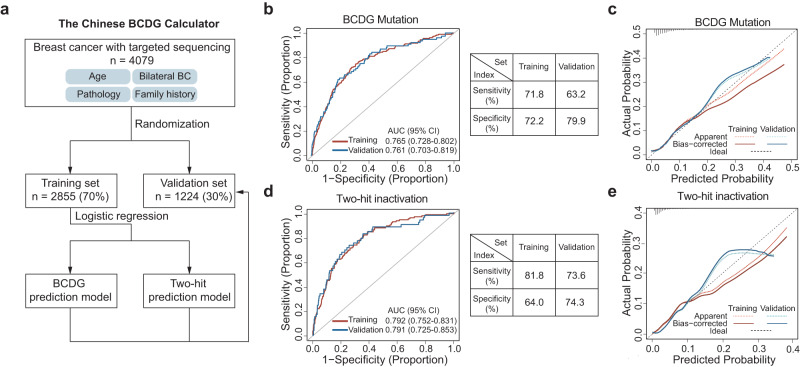
Establishment of a population-specific BCDG germline mutation prediction model. **a** Workflow of establishing predictive models for BCDG mutation and two-hit inactivation. **b** ROC curves of the BCDG mutation prediction model in the training set (red) and validation set (blue). **c** Calibration plots of the predicted and actual probabilities of the BCDG mutation model in the training set (red) and validation set (blue). **d** ROC curves of the two-hit inactivation prediction model in the training set (red) and validation set (blue). **e** Calibration plots of the predicted and actual probabilities of the two-hit inactivation model in the training set (red) and validation set (blue).

Based on the above findings, appropriate strategies are urgently needed to identify BCDG mutations and two-hit status. Therefore, through two logistic regression models, the Chinese BCDG Calculator was established and validated on the basis of the FUSCC-BC cohort to predict BCDG mutations and their two-hit status (Fig. 7a). The Chinese BCDG Calculator incorporates age at diagnosis, pathological features, bilateral breast cancer, FBOC, and family history of other cancers as predictors. The area under the curve (AUC) of the prediction model for BCDG mutations was 0.765 (sensitivity = 71.8%; specificity = 72.2%) in the training set and 0.761 (sensitivity = 63.2%; specificity = 79.9%) in the internal validation set (Fig. 7b). The calibration of the prediction model was evaluated with the calibration curves in both the training and internal validation sets, and the Hosmer–Lemeshow (HL) test suggested that there was no significant departure from a perfect fit (*P* = 0.471 and *P* = 0.533, respectively; Fig. 7c). Similarly, another model for predicting two-hit inactivation also showed good performance in the training set (AUC = 0.792, 95% CI: 0.752–0.831; sensitivity = 81.8%; specificity = 64.0%) and internal validation set (AUC = 0.791, 95% CI: 0.725–0.856; sensitivity = 73.6%; specificity = 74.3%; Fig. 7d), both of which were calibrated (HL *P* = 0.704 and HL *P* = 0.401, respectively; Fig. 7e). Despite the potential higher accuracy of the model for predicting two-hit inactivation, it pertains to a smaller subset of individuals. Taken together, the ethnicity-specific customization of mutation prediction models is critical for familial genetic risk assessment and can guide treatment decisions in the Chinese population.

## Discussion

Germline-somatic mutation interactions have been recognized as key contributors to various biological processes in cancer progression. However, their specific role in breast cancer, particularly in East Asian populations, remains poorly understood. In this study, we leveraged the largest targeted sequencing cohort of paired tumor-blood genomic data and conducted a systematic analysis, shedding light on the comprehensive genomic profiling of breast cancer genomes and the impact of germline-somatic mutation interactions on tumorigenesis and clinical outcomes. Our findings demonstrated distinct patterns of somatic and germline mutations in the Chinese population compared to the Caucasian and African populations. Furthermore, we identified five inherited breast cancer DNA repair-associated genes dubbed as BCDGs and established that two-hit inactivation in BCDGs is associated with significant clinical benefits from platinum-based therapies, PARPi, and ICIs. In addition, leveraging our TNBC multiomics cohort, we uncovered that improved clinical benefits from DNA damaging-targeted therapy and immunotherapy are associated with increased genomic instability and an immune-activated TME, respectively. Finally, the development of the Chinese BCDG Calculator is a crucial step in accurately predicting BCDG mutations and two-hit inactivation, providing valuable insights for genetic counseling and treatment decision-making.

A growing number of studies have reported somatic mutations in breast cancer in different races. However, most research has focused on profiling data for females of Caucasian ancestry^36–39^, while Asians continue to be underrepresented. In our study, the somatic mutation differences between Chinese and Caucasian populations were primarily concentrated in the HR^+^/HER2^–^ subtype, such as an increased frequency of *NF1* and *TP53* mutations in the Chinese population. Regarding the disparities between Asians and Africans, we observed an increased *PIK3CA* mutation and a decreased *GATA3* mutation in our cohort. Such a difference should be interpreted cautiously since many of the clinicopathological factors, such as clinical subtype, are not available in the GENIE cohort. Consistently, it has been demonstrated that the African population also exhibits a lower *PIK3CA* mutation and a higher *GATA3* than Caucasians^36,39^. Collectively, we revealed racial disparities in somatic mutation profiles, providing valuable clues for further investigation into tumorigenesis and therapeutic potential.

Despite the well-established consensus on the value of genetic testing in breast cancer^40^, determining which genes should be prioritized for testing, especially in the Chinese population, remains uncertain. To address this uncertainty, we performed a case‒control study and identified five BCDGs, including *BRCA1*, *BRCA2*, *CHEK2*, *PALB2*, and *TP53*. Similar to the Caucasian population, our study demonstrated that 4.7% of patients carried *BRCA1* or *BRCA2* variants. The identification of potential ethnicity-specific founder mutations in *BRCA1/2* is a crucial step in the advancement of genetic counseling, as it enables a more targeted approach to genetic testing. The most well-known example of a founder effect is the Ashkenazi Jewish population, which harbors three founder mutations in *BRCA1* (E23fs and Q1756fs; Fig. 3d) and *BRCA2* (S1982fs)^41^. Interestingly, we identified three recurrent *BRCA1* variants: p.T327fs (0.98‰), p.N704fs (0.98‰) and p.I1824fs (4.66‰). A previous study identified *BRCA1* p.I1824fs in the Han population as a founder mutation^42^, while *BRCA1* p.T327fs and p.N704fs could potentially be novel founder mutations in the Chinese population, warranting further investigation.

Additional breast cancer susceptibility genes, including *CHEK2*, *PALB2*, *ATM*, *PTEN*, and *TP53*, have been identified in Caucasian and African populations^43,44^. In *BRCA1/2*^−^ breast cancer, *PALB2* germline variants were the most prevalent. The prevalence of *PALB2* mutations appears to be relatively high within East Asians, with distinct recurrent mutations observed among various races^45,46^. For example, the Finnish founder mutation *PALB2* 1592delT was not detected in our population. Although the frequency of *CHEK2* in our cohort was similar to that in the TCGA cohort^18^, *CHEK2* c.1100delC and p.Ile157Thr, two well-characterized Caucasian founder mutations, were absent in the Chinese population^47,48^. In addition, *CHEK2* variants are not related to an earlier age of onset in the FUSCC cohort but are associated with a younger onset age in patients of Caucasian ancestry^49,50^. In our study, neither *ATM* nor *PTEN* was significant in the overall case‒control analyses, while they were determined to be statistically significant in the case‒control analysis of the HR^+^/HER2^–^ subtype. The main possible cause of this was the fact that the cohort was not large enough. To elucidate the breast cancer risk for *ATM* and *PTEN*, additional broader case‒control studies or family-based segregation analyses are required in the Chinese population. Interestingly, we discovered that the *TP53* gene increased the risk of breast cancer in Chinese patients and that germline *TP53* carriers had an earlier age at diagnosis, especially those with the HER2^+^ subtype. However, we also observed *TP53* germline mutations leading to breast cancer in one elderly woman. This suggests that germline mutations are not the sole factor in the early onset of breast cancer. Instead, it is a combination of germline genetic variations, somatic acquired mutations, and environmental exposures^1,51^. Nevertheless, two recent population-based case‒control studies among Caucasians indicated that the *TP53* gene was not a breast cancer predisposition gene^52,53^. One possible explanation for the discrepancy was the fact that the frequency of germline *TP53* variants in East Asian breast cancers was higher than that in Caucasians. Although no association between *MUTYH* variants and breast cancer risk has been established, there is a high prevalence of these variants in East Asians. Notably, the *MUTYH* variants, especially the recurrent W153X variants, conferred sensitivity to platinum treatment. Similar to the pathological features of Caucasian populations with germline high-risk gene mutations^50,54^, BCDG carriers show distinct clinicopathological features, such as a younger age of onset, a higher Ki67 index, more advanced disease staging, and higher frequencies of bilateral breast cancer and FBOC. Overall, we identified an ethnicity-specific panel of five BCDGs and found their potential associations with clinicopathological features.

Germline-somatic mutation interactions are a key element involved in tumorigenesis^1,55^. Therefore, we leveraged a unique cohort to systematically investigate the role of germline-somatic mutation interactions in breast cancer. Co-occurrence and mutual exclusivity between germline and somatic mutations of different driver genes, as well as two-hit inactivation of the same driver genes constituted the main types of germline-somatic mutation interactions. Furthermore, our research not only validated previous findings about some of the co-occurring and mutually exclusive mutations^30^ but also showed several significant insights: (1) molecular subtypes of breast cancer were validated by the relationship between germline *BRCA1*/*BRCA2*/*ATM* variants and recurrent *TP53* and uncommon *PIK3CA*/*GATA3* somatic aberrations; and (2) germline *MUTYH* variants were found to co-occur with *BRCA1* somatic mutations, which may indicate that *MUTYH* and *BRCA1* have a potential synergistic effect on the occurrence and therapeutic efficacy of breast cancer. Collectively, we revealed that there were indeed interactions between germline and somatic alterations, especially in BCDGs. Further investigation into their effects on disease progression and treatment response is needed.

Previous research has shown that allelic imbalance may be a novel class of candidate biomarkers of drug response^10^. Therefore, we utilized matched tumor-blood genomic data, coupled with detailed clinical annotation, to systematically elaborate the biological impacts and clinical associations of two-hit events in Chinese breast cancers. Further analyses showed that BCDG carriers, particularly those with two-hit inactivation, had a younger age of onset, a higher Ki67 index, and a higher TMB than noncarriers. We next investigated the impact of two-hit inactivation on treatment response in the setting of clinical trials. Remarkably, BCDG carriers with two-hit inactivation had better treatment responses and improved survival outcomes when treated with platinum-based chemotherapy than noncarriers. Interestingly, *BRCA1/2* carriers with two-hit inactivation obtained better treatment responses and clinical outcomes from PARPi therapy than monoallelic carriers. To explore the mechanisms behind improved treatment outcomes of platinum and PARPi in two-hit tumors, we leveraged the FUSCC-TNBC multiomics cohort to reveal the characteristics of two-hit tumors. We observed that these tumors not only exhibit an increased mutational load but also show a greater contribution of mutational signature 3. Furthermore, they have higher scores for HRD and CIN, indicating elevated levels of genome instability. Additionally, we found that two-hit tumors display upregulated signaling of DNA double-strand break repair via homologous recombination. These findings suggest that the better responses observed in two-hit tumors may be attributed to the genomic instability and lack of homologous recombination competence in two-hit tumors. Importantly, this interesting finding could be used to optimize patient selection for PARPi. However, our result contradicted previously published literature suggesting a similar clinical benefit from PARP inhibition for both heterozygous and biallelic patients with BRCA-associated cancer types^56,57^. Multiple studies have noted that the effect of *BRCA1/2* zygosity on the HRD phenotype is lineage-specific, indicating the need for further investigation into the relationship between zygosity and the response to PARPi. Interestingly, Fabrice Andre et al. reported that patients with increased HRD or loss of heterozygosity on *BRCA1/2* derive a high benefit from olaparib^58^. Moreover, biallelic *BRCA1* mutation carriers also have a trend toward better responses and improved PFS outcomes when treated with ICIs. One possible explanation for this is that two-hit carriers have a higher TMB, resulting in enhanced presentation of immunogenic neoantigens through MHC class I molecules^59,60^. Additionally, the interactions between *BRCA1/2* mutations and the TME, involving NK cells, macrophages, and dendritic cell populations, may also contribute to these observations^59,61^. Ultimately, we revealed an association between two-hit inactivation and clinicopathological phenotypes, suggesting that two-hit inactivation in BCDG may confer sensitivity to platinum agents, PARPi, and ICIs.

Based on the important findings mentioned earlier, we developed and validated two predictive models using our cohort to identify BCDG mutations and determine their two-hit status. Through multivariate regression analyses, we identified significant risk factors associated with BCDG mutations, which included age at diagnosis, Ki67 index, TNBC, history of contralateral disease, and family history of cancer. Thus, the established Chinese BCDG Calculator incorporated age at diagnosis, pathological features, bilateral breast cancer, FBOC, and family history of other cancers as predictors. These robust predictive models are essential for genetic counseling purposes and can aid in the selection of patients with two-hit inactivation in BCDG who are more likely to benefit from targeted therapies. This is especially valuable in regions where targeted sequencing is not readily available.

Our study has several limitations that need to be addressed in future investigations. First, although our study included the largest sequencing cohort for studying the breast cancer genome, the sample size for evaluating specific drug-gene interactions was still quite modest. Therefore, these findings require further validation in larger cohorts, particularly those involving PAPRi and immunotherapies. Second, the association between two-hit inactivation in BCDG and clinical outcomes was established through retrospective analysis, and its validation in prospective clinical trials is warranted. Finally, future research should delve into more detailed molecular mechanisms to enhance our understanding.

In conclusion, our study utilized a large-scale targeted sequencing cohort and a multiomics cohort to investigate the association between germline-somatic mutation interactions, treatment outcomes, and the underlying mechanisms. We found that germline-somatic mutation interactions, particularly two-hit inactivation, were associated with clinical benefits from DNA damaging-targeted therapy and immunotherapy. The Chinese BCDG Calculator will facilitate risk management, prognostic estimation, and treatment decisions for Asian breast cancer patients in the future. Moreover, this clinical cohort not only serves as a large database of breast cancer genomes but also provides a crucial reference for further advancements in precision oncology.

## Materials and methods

### Patients, specimens and clinical data

Our program utilized data from two large cohorts to explore germline-somatic mutation interactions and elucidate their role in treatment outcomes as well as the underlying mechanisms.

Cohort 1 (FUSCC-BC) is a prospective clinical sequencing cohort. A total of 4079 consecutive Chinese breast cancer patients treated at the Department of Breast Surgery at Fudan University Shanghai Cancer Center (FUSCC) between April 2018 and June 2021 were enrolled according to the following defined criteria: (1) female patients diagnosed with unilateral invasive breast carcinoma; (2) pathological examination of tumor samples examined at the Department of Pathology of FUSCC (estrogen receptor (ER), progesterone receptor (PR), and HER2 statuses individually evaluated by two experienced pathologists based on IHC analysis and fluorescence in situ hybridization) — tumor specimens were classified into breast cancer subtypes based on ER and PR status and the HER2 IHC and/or FISH results rendered at the time of diagnosis according to the American Society of Clinical Oncology and College of American Pathology guideline recommendations^62,63^; and (3) sufficient fresh tumor tissue available for further research. The clinicopathological characteristics included age, histological tumor type, tumor size, lymph node status, histological grade, therapy, and ER, PR, HER2, and Ki67 status. We initiated multiple umbrella trials to practice genomics-guided precision treatment in HR^+^/HER2^–^ or TNBC patients, including the FUTURE trial (NCT03805399), the FUTURE-CPLUS trial (NCT04129996), and the MULAN trial (NCT04355858). All tumor and peripheral blood samples included in the present study were obtained after the research study was approved by the FUSCC Ethics Committee. The Clinical Research Ethics Committee of Fudan University Shanghai Cancer Center approved both the research protocol and the patients’ informed consent. Family history information was ascertained through medical record review, including inpatient and outpatient visit records and telephone interviews. A family history of cancer was defined as having at least one first- or second-degree relative with malignant tumors. Follow-up within this cohort of patients was updated until April 2022 via medical records and phone contact.

Cohort 2 (FUSCC-TNBC) is a multiomics cohort consisting of Chinese TNBC patients treated at the Department of Breast Surgery at FUSCC from January 2007 to December 2014. In this study, we selected all 313 patients who qualified to undergo zygosity estimation. All tissue samples included in this study were also obtained with the approval of the FUSCC Ethics Committee and written informed consent from each patient.

### Data generation for the FUSCC-BC and FUSCC-TNBC cohorts

FUSCC-BC cohort: The tumor specimens were transferred to the Chinese National Human Genome Center at Shanghai for deep-coverage sequencing. All sequencing data were further analyzed and uploaded in the database within 3 weeks. A total of 4079 matched tumor and normal DNA pairs were collected at FUSCC. Genomic sequencing was performed on tumor DNA extracted from fresh-frozen tumor biopsy samples and normal DNA extracted from mononuclear cells from peripheral blood using TGuide M24 (Tiangen, Beijing, China). The purity and quantity of the total DNA were evaluated by measuring the absorbance at 260 nm (A260) and 280 nm (A280) using a NanoDrop 2000 spectrophotometer (Thermo Scientific, Wilmington, DE, USA). The extracted DNA was considered pure and appropriate for further experiments if the A260/A280 ratio was within the 1.6–1.9 range.

All tumor samples underwent next-generation sequencing in our laboratory using the FUSCC-BC panel, which included the 484-gene version 1 panel and 539-gene version 2 panel (Supplementary Table S10). The panel was designed to detect mutations, small indels, and copy number alterations. In-house-generated RNA baits were used to capture all protein-coding exons of the target genes. The RNA baits were produced from an oligo pool synthesized by Synbio Technologies (Suzhou, China). The oligo pool was converted into double-stranded DNA. The T7 promoter site was integrated into the amplicon, and the DNA was transcribed into biotinylated RNA. Subsequently, the biotinylated RNA was purified, quantified, and used for target enrichment.

The tumor and matched normal blood samples were simultaneously sequenced. Over 10 ng of each DNA sample obtained after SYBR green quantification was fragmented using a Covaris M220 and then subjected to terminal repair, A-tailing and adapter ligation were performed using a KAPA HyperPlus kit (Kapa Biosystems) based on the manufacturer’s recommended protocol. Then, prepared DNA (750 ng) in a volume of 3.4 μL was captured by RNA baits, and the captured library was purified and amplified with index primers. After quantification with a Multi-Mode Reader (BioTek), the libraries were pooled and sequenced utilizing an Illumina HiSeq X TEN platform (Illumina Inc., San Diego, CA, USA). For the collection of genomic data, targeted sequencing was performed on the Illumina NovaSeq 6000. Bases were called using the following software from Illumina: NovaSeq control software v1.7.5 and Illumina bcl2fastq v2.20. The variant calling and coverage analysis of each capture region were examined using an in-house bioinformatics pipeline according to the general variant calling pipeline. Reads were aligned to the human reference genome (GRCh37/hg19) utilizing the Burrows–Wheeler Aligner (BWA, v0.7.17-r1188) with the BWA-MEM algorithm and default parameters. The Genome Analysis Toolkit (GATK, v4.0.1.2.0) was utilized to locally realign the BAM files at intervals that included indel mismatches and recalibrate the base quality scores of the reads in the BAM files.

Details regarding the generation of DNA sequencing data for the FUSCC-TNBC cohort are described in the Methods section as previously reported^3^.

### Somatic variant calling

GATK Mutect2 was used to identify somatic mutations. The VCF files were annotated using ANNOVAR (v2015-06-17). The variants and annotation results were transferred to Excel spreadsheets. A panel of normal (PoN) samples was used to screen out expected germline variations and artifacts to improve specificity. Each alteration identified by the pipeline was manually reviewed to confirm that no false-positive variants were reported. SAMtools (v2.6.2) and GATK were used to acquire the sequencing quality statistics. The FACETS algorithm (v0.16.0) was used to detect gene-level amplification and deletion^64^.

### Curation of DDR pathway genes

A list of 62 DDR genes was compiled from pertinent gene lists (Supplementary Table S5), including the online table of DDR pathway genes (https://www.mdanderson.org/documents/Labs/Wood-Laboratory/human-dna-repair-genes.html) and the curated catalog of DDR genes from previously published literature^24,65^.

### Cancer predisposition gene selection and penetrance stratification

A set of 78 genes was assembled for further germline analysis based on four main resources (Supplementary Table S11): (1) the American College of Medical Genetics (ACMG) gene list^66^; (2) the Pathogenicity of Mutation Analyzer (PathoMAN) gene list^67^; (3) the Characterization of Germline Variants (CharGer) gene list^18^; and (4) the curated cancer susceptibility genes from the previously published literature by Rahman^68^ and others. In addition, these 78 cancer predisposition genes were categorized into two tiers: (1) tier one genes were related to hereditary breast cancer, and (2) tier two genes were associated with other hereditary malignancies. The genes with pathogenic germline variants were classified into one of five categories based on known disease risks and prior modeling^69^: high penetrance (relative risk (RR), > 5), moderate penetrance (RR, 2–5), low penetrance (RR, < 2), uncertain penetrance for genes with pathogenic germline variants that are not well characterized, and autosomal recessive conditions. RR estimates were compiled from a literature review per the National Comprehensive Cancer Network clinical practice guidelines for breast, ovarian, and/or pancreatic cancer genetic assessment (http://www.nccn.org) and reviewed by our medical geneticists from FUSCC to validate penetrance categories for each gene.

### Germline variant identification and annotation

GATK HaplotypeCaller was used to identify germline SNVs and germline indels. Only variants with high confidence were retained in accordance with the following criteria: for protein-altering and splice site variants, (1) at least 20× coverage, (2) allelic depth (AD) ≥ 10 for the alternative allele, and (3) variant allele frequencies ≥ 30%.

Germline variants of 78 cancer predisposition genes classified as pathogenic or likely pathogenic by both InterVar^70^ and CharGer^18^ were considered P/LP variants. Inconsistent annotations between the two programs were manually resolved by reviewing the ClinVar database. Only variants classified as P/LP were retained. Inconsistent annotations between InterVar and CharGer were also reannotated with a third program, PathoMAN^67^, to check the assigned ACMG criteria of all three programs. The inconsistency was manually resolved by a literature review to determine the pathogenicity. Finally, the list was manually reviewed to remove the variants inconsistent with the role of the genes (for example, inactivating mutations in oncogenes). The detailed germline mutation calling and filtering procedures are shown in Supplementary Fig. S3a.

The control dataset was obtained from the Genome Aggregation Database (GnomAD) (http://gnomad.broadinstitute.org/)^71^, which included whole-exome (WES) or whole-genome sequencing data from 141,456 unrelated individuals. Low-quality variants in GnomAD were excluded. Considering ethnic specificity, the East Asian populations (*N* = 9977) in GnomAD were also collected separately. The pathogenicity of alterations in the reference controls was annotated by the same pathogenicity prioritization pipeline as ours.

### Examination of co-occurrence and mutual exclusivity between germline and somatic alterations

A list of cancer driver genes and functional alterations was first assembled in two steps: First, we assembled a list of oncogenes (OGs) and tumor suppressor genes (TSGs) with five resources: (1) cancer-related genes annotated as OG or TSG by Cancer Gene Census^72^; (2) the set of cancer driver genes from OncoKB^73^; (3) the curated cancer genes from ten canonical signaling pathways^74^; (4) a compendium of mutational driver cancer genes from the previously published literature by Martinez-Jimenez et al.^75^; and (5) the functionally validated mutational driver cancer genes^76^. Contradictory annotations among five sources were manually resolved.

All functional alterations were then determined for the examination of co-occurrence and mutual exclusivity. Regarding TSGs, truncating mutations (nonsense mutations, splicing mutations and frame-shift insertions or deletions) were classified as putative functional. For both TSGs and OGs, known cancer hotspot mutations and oncogenic or likely oncogenic mutations were also retained^74,76–79^. Hotspot mutations in the Cancer Hotspots database were annotated using the annotateMaf R package (github.com/taylor-lab/annotateMaf), and oncogenic or likely oncogenic mutations in the OncoKB database were annotated using oncokb-annotator (github.com/oncokb/oncokb-annotator). In addition, functional mutations computationally predicted based on the dbNSFP database were retained^78^. Finally, the list was manually reviewed to remove the variants inconsistent with the role of the genes (for example, inactivating mutations in oncogenes). A flowchart of annotations for functional alterations is exhibited in Supplementary Fig. S6a. The list of cancer driver genes is provided in Supplementary Table S12.

After the selection of somatic functional alterations and germline pathogenic variants, we used the somaticInteractions function from the maftools R package to estimate the co-occurrence and mutual exclusivity to indicate germline-somatic mutation interactions.

### Two-hit event analysis

We inferred somatic zygosity for germline pathogenic variants based on tumor purity, locus-specific and allele-specific DNA copy-number inference, and the observed VAF in the tumor^1,10,57^. Germline variants were determined to be heterozygous, two-hit inactivated or to have lost the mutant allele. To determine whether a germline variant is in ASI in the corresponding tumor sample, we compared whether the observed VAF was consistent with the expected VAF given the tumor purity and local copy number calculated as (ф × LCN + (1 – ф))/(ф × MCN + 2 × (1 - ф)), where ф is the tumor purity, and LCN and TCN are the lesser and total copy number, respectively. Germline variants were determined to be heterozygous if the observed VAF was either (1) concordant with the expected VAF (within the 95% binomial CI) given balanced heterozygosity or (2) less than the lower bound of the 95% CI of the expected VAF given genomic gains. The observed VAF for germline variants with ASI status was within the 95% CI (or greater than the 95% CI) of the expected VAF corresponding to an observed copy number state other than balanced heterozygosity. Another form of two-hit inactivation is a pathogenic germline variant coupled with a missense or truncating somatic mutation in the same gene^1,18^. Loss of the WT allele was deemed present if its observed VAF was either within the 95% CI or greater than the lower bound of the 95% CI of the expected VAF of the LCN having a copy number of 0. In contrast, loss of the mutant allele was determined to be the opposite of the above second scenario. The two-hit event of the germline variant was considered indeterminate if (1) TCN and LCN derived from FACETS were not evaluable and (2) the germline variant was homozygous.

### Computation of HRD scores

The HRD score was obtained by summing three scores: allelic imbalance extending to the telomere (NtAI), loss of heterozygosity (LOH), and modified large-scale state transition (LSTm)^80^. The computation method for these three scores has been previously described and summarized by Telli and colleagues. To minimize the impact of ploidy on the LST score, the formula LSTm = LST – 15.5 × ploidy was utilized.

### Estimation of neoantigens

Using the WES data (.bam) of paired normal samples from TNBC patients, we inferred the 4-digit HLA genotype for each sample using the POLYSOLVER tool^81^. The somatic mutation data (.maf) and HLA genotype data were used as inputs for the prediction of neoantigens using NetMHCpan (v4.0)^60^. Neoantigens derived from protein-coding SNVs and indels were predicted independently. As neoantigens, mutations that were predicted to generate peptides with an affinity less than 500 nM and whose corresponding gene was expressed above Combat value 1 were selected. We referred to pVAC-seq^82^ and made some modifications based on the features of our dataset to construct this algorithm.

### Mutational signature

Using multivariate analysis and the “SigMA” R package (github.com/parklab/SigMA), the mutational Signature 3 status was predicted^83^. The final Signature 3 score, which combines likelihood with cosine similarity and exposure of Signature 3 obtained using the non-negative least-squares (NNLS) algorithm, was generated and then compared to tumor type-specific thresholds. We executed “SigMA” with the following parameters: tumor_type = “breast” and data = “seqcap”.

### Analysis of RNA sequencing data

The generation of RNA sequencing data for the FUSCC-TNBC cohort was described in our previous study^3^. Based on bulk RNA-seq data, immune-related signatures, such as MHC class I^84^ and TMEscore^85^, were evaluated using the “IOBR” R package^86^. Additionally, we employed CIBERSORT^87^ to calculate an immune infiltrate score and identified differentially expressed genes using the “Limma” R package^88^. Pathway enrichment analysis and GSEA were performed using the ClusterProfiler R package (v3.18.1). ssGSEA (“GSVA” function in R) was used to estimate the enrichment of each DNA damage repair pathway for each patient^89^.

### Development and validation of the BCDG prediction model and two-hit prediction model

The study sample was randomly split into training and validation sets, comprising 70% and 30% of the samples, respectively. Based on the previous analysis, candidate predictors included age at diagnosis, bilateral breast cancer, pathological features (including histological grade, tumor size, lymph node status, distant metastasis, ER status, PR status, HER2 status and Ki67), family history of breast cancer (FHBC), family history of ovarian cancer (FHOC), and family history of other cancers. Missing data in the training and validation sets were imputed using multiple imputations by chained equations under the missing at random assumption. A BCDG or two-hit inactivation prediction model was built based on the logistic regression method using the training set and tested in the validation set. Model calibration and discrimination were evaluated in the training validation sets using the Hosmer–Lemeshow test and AUC, respectively. The risk score _(BCDG)_ was calculated as follows: –1.561 – 0.039 × age + 0.094 × grade – 0.079 × T + 0.133 × N – 0.030 × M + 0.277 × ER – 0.290 × PR – 1.599 × HER2 + 0.017 × Ki67 + 0.949 × contralateral breast cancer + 1.074 × FHBC + 1.515 × FHOC + 0.320 × other cancer history. The risk score _(two-hit)_ = –2.371 – 0.038 × age – 0.055 × grade + 0.087 × T – 0.032 × N – 0.228 × M + 0.166 × ER – 0.185 × PR – 1.879 × HER2 + 0.021 × Ki67 + 0.938 × contralateral breast cancer + 1.045 × FHBC + 1.075 × FHOC + 0.506 × other cancer history. The probability threshold of each model was chosen based on the intersection of the sensitivity and specificity curves.

### Cell lines and culture

The cell lines HEK293T, Hs578T, LM2-4175 and T47D were obtained from the cell bank of the Type Culture Collection of the Chinese Academy of Sciences (Shanghai, China). The cell lines were monitored for mycoplasma contamination and verified by short tandem repeat profiling. Targeted sequencing showed no endogenous *MUTYH* mutations in the three different WT cell lines (T47D, Hs578T, and LM2-4175). The DNA sequencing data of the three cell lines have also been uploaded to the NCBI Sequence Read Archive (SRA) database under accession code SRP406549. These data can be searched on the SRA website (http://www.ncbi.nlm.nih.gov/sra) by pasting the accession code into the text search box or through the following hyperlinks: https://trace.ncbi.nlm.nih.gov/bioproject/898860. The cells were incubated in DMEM supplemented with 10% fetal bovine serum (ExCell Biol, No. FSP500), 50 U/mL penicillin and 50 μg/mL streptomycin (BasalMedia, No. S110B). All cells were cultured in a 5% CO_2_ incubator at 37 °C.

### DNA constructs and viral transduction

The plasmids encoding WT *MUTYH*, mutant *MUTYH* W153X, and mutant *MUTYH* Q265X were purchased from Shanghai GeneChem and subcloned into the lentiviral vector GV350-Ubi-MCS-3FLAG-SV40-Neomycin according to the standard protocol. All construct sequences were verified by Sanger sequencing at Sangon Biotech (Shanghai, China). The DNA constructs were transfected into HEK293T cells using Neofect DNA transfection reagent (Tengyi Biol; No. TF201201). The supernatants containing the virus were collected after 48 h of transfection, filtered, and used to infect cells in the presence of 8 μg/mL polybrene (Sigma; No. H9268). After 48 h of infection, cells were selected for one week with 0.5 mg/mL G418 (Sangon Biotech; No. A600958-0005). Gene overexpression efficiency levels were validated by western blotting assay.

### Antibodies and immunoblotting assay

For immunoblotting, the cells were lysed in RIPA buffer containing protease inhibitors (Bimake; No. B14002) and phosphatase inhibitors (Bimake; No. B15003). Protein concentrations were detected by bicinchoninic acid assay (Yeasen Biotechnology; No. 20201ES90). Then, the proteins were resolved by SDS-PAGE gel and transferred to PVDF membranes (EMD Millipore; No. IPVH00010). After blocking with 5% bovine serum albumin (Sigma; No. V900933-1KG), the membranes were incubated at 4 °C overnight with the indicated primary antibodies, including Flag (1:5000, Sigma; No. GNI4110-FG), MUTYH (1:1000, Abcam; No. ab228722) and Vinculin (1:3000, Sigma; No. V9131). After washing with PBS three times the next day, the membranes were incubated with horseradish peroxidase-conjugated mouse or rabbit secondary antibodies (1:5000, Cell Signaling Technology; No. 7076V and 7074V, respectively). Finally, the protein bands were visualized using an enhanced chemiluminescence substrate kit (Yeasen Biotechnology; No. 36208ES80).

### IC_50_ and cell colony formation assays

For the IC_50_ assays, 4 × 10^3^ cells were plated in 96-well plates overnight and then treated with the indicated concentrations of cisplatin for 72 h. Cell viability was measured using a CCK-8 kit (Yeasen; No. 40203ES92) by adding 10 μL CCK-8 solution to each well, and the absorbance at 450 nm (A450) was measured at the indicated times. The IC_50_ was calculated using GraphPad Prism. Colony formation assays were carried out in a 6-well plate (1000 cells per well). After 24 h, the cells were treated with the indicated concentration of cisplatin. The medium supplemented with the indicated concentration of cisplatin was changed every three days. After 14 days of culture, the cells were fixed with methanol, stained with crystal violet, and counted.

### Xenograft mouse models and treatment

All procedures were conducted in accordance with institutional guidelines for the Care and Use of Laboratory Animals and were approved by the Animal Experiments Committee of Fudan University Shanghai Cancer Center (FUSCC-IACUC-2022088). A total of 1.5 × 10^6^ LM2-4175 cells were inoculated into the mammary fat pads of 6-week-old BALB/c nude female mice. When the average tumor volumes reached 100 mm^3^, PBS or cisplatin (3 mg/kg) was administered by intraperitoneal injection every 2 days for 6 times. Tumor sizes were examined every 3 days and calculated as tumor volumes = (length × width^2^)/2. Once the mice were euthanized, the tumors were removed and weighed.

### Statistical analyses

Summary statistics, including mean values, median values, interquartile ranges, and frequency tabulation, were used to characterize the data distribution. Student’s *t-*test, the Mann–Whitney Wilcoxon test and the Kruskal–Wallis test were performed for comparisons of continuous variables and ordered categorical variables, while Pearson’s chi-square test and Fisher’s exact test were utilized to compare unordered categorical variables. The *P* values were adjusted by using the FDR or the Bonferroni–Hochberg correction for pairwise comparisons. A multivariate logistic regression model was used to adjust covariates in the comparison analyses. The predictive factors for germline mutations were identified by multivariate logistic analysis. DFS was defined as the date of surgery to the date of the first recurrence or contralateral breast cancer occurrence, whereas PFS was determined from the salvage treatment date until progression (for PFS) or the last follow-up. The association between mutation status and survival was evaluated using the Kaplan–Meier method and Cox regression models. All analyses were performed using R package version 4.0.3 (R Foundation, Vienna, Austria). Unless otherwise noted, a *P* value < 0.05 was considered statistically significant.

### Supplementary information

Supplementary figures

Supplemenatry tables

## Data Availability

The DNA sequencing data in our FUSCC-BC cohort have been deposited in The National Omics Data Encyclopedia (NODE) (http://www.biosino.org/node), which is a publicly accessible database from the Chinese Academy of Sciences. All data can be viewed by searching with the accession numbers (OEP001027 and OEP003469) or through the URLs: http://www.biosino.org/node/project/detail/OEP001027 and http://www.biosino.org/node/project/detail/OEP003469. The data that support the findings of this study have been uploaded to the Fudan Data Portal (https://data.3steps.cn/cdataportal/study?id=FUSCC_BRCA_panel_4000). All raw data from the FUSCC-TNBC cohort can be accessed in NODE and viewed by searching with the accession number OEP000155 or through the URL: http://www.biosino.org/node/project/detail/OEP000155. Sequencing information can also be found in SRP157974 (WES and RNAseq) and GSE118527 (OncoScan). All the other data supporting the findings of this study are available within the article and its Supplementary information files and from the corresponding author upon reasonable request. Specific codes will be made available upon request to Z.-M.S.
