# Supplementary material for: Comprehensive genomic profiling of breast cancers characterizes germline-somatic mutation interactions mediating therapeutic vulnerabilities
Source: Cell Discov. 2023 Dec 19;9:125. doi: 10.1038/s41421-023-00614-3 (PMC10730692; doi:10.1038/s41421-023-00614-3)
Supplement: Supplementary file 1 — Supplementary figures [file 41421_2023_614_MOESM1_ESM.pdf]

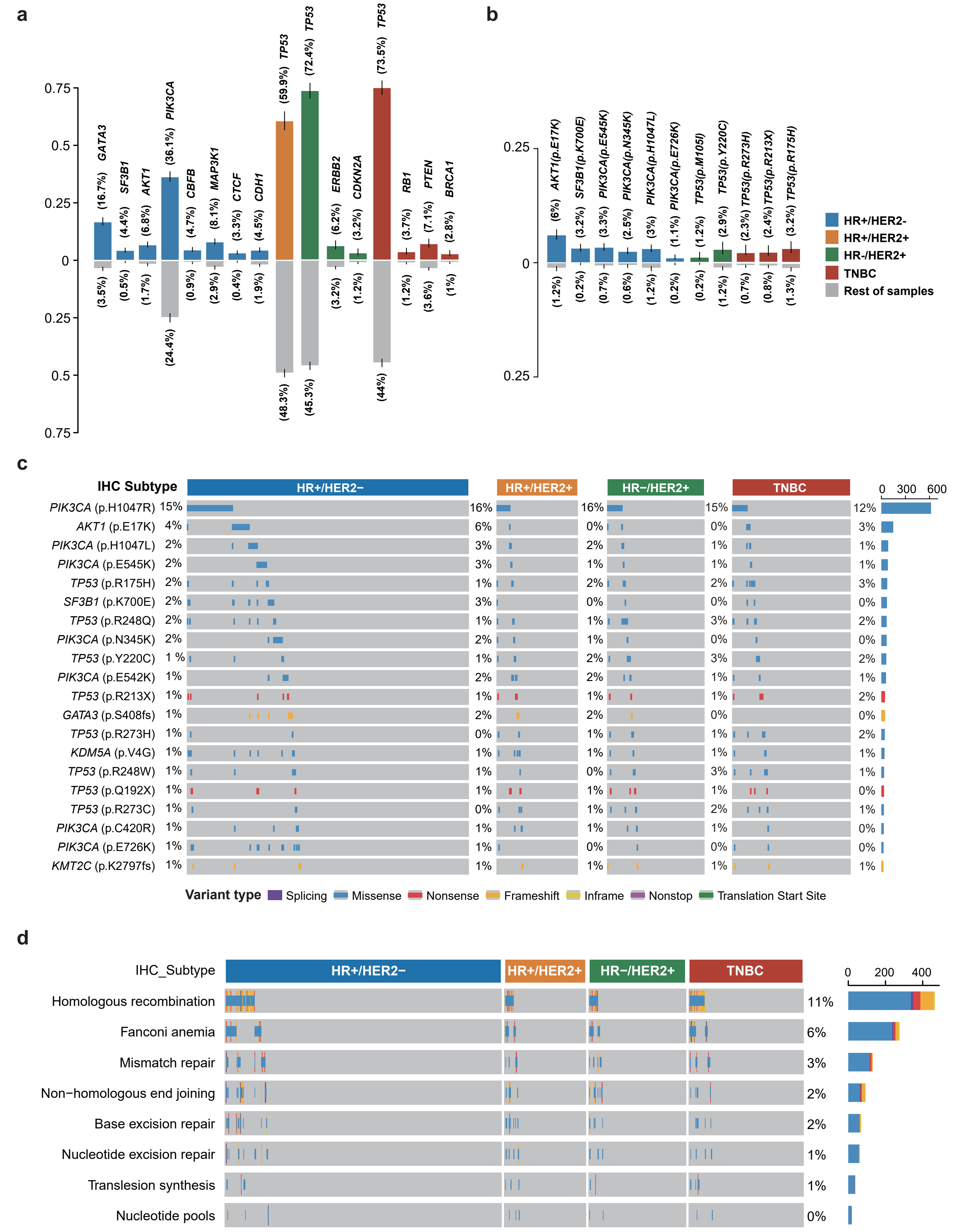

Supplementary Fig. S1. The genomic characteristics of Chinese breast cancer. a Significant enrichment of genomic mutations in different molecular subtypes. **b** Significant enrichment of mutation hotspots in distinct subtypes of breast cancer. **c** Landscape of mutation hotspots categorized by the molecular subtype and annotated with both the variation type and mutation frequency. The mutation counts in each mutation hotspot are presented on the right side. **d** The landscape of DDR pathway mutations in 4079 samples categorized by the molecular subtypes and annotated with both the variation type and mutation frequency. The mutation counts in each DDR pathway are presented on the right side.

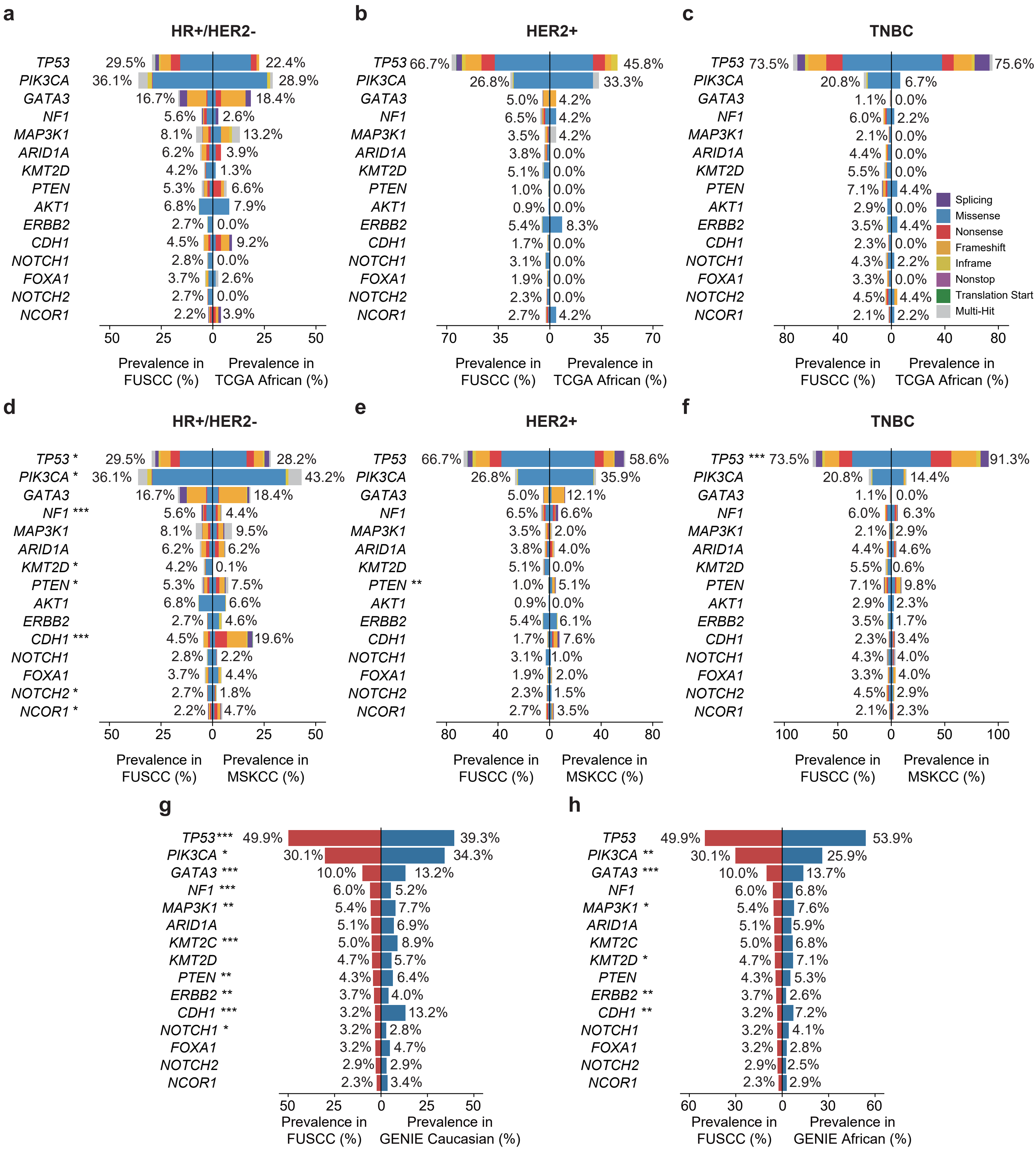

Supplementary Fig. S2. Ethnicity-specific genomic mutations compared with TCGA, MSKCC, and GENIE cohorts. a-c Mutation frequency comparison with TCGA African cohort in each molecular subtype. Logistic regression accounts for age and histology. **d-f** Mutation frequency comparison with MSKCC cohort in each molecular subtype. Logistic regression accounts for age, histology, sample site, and treatment. **g,h** Mutation frequency comparison with GENIE Caucasian or African cohorts. Logistic regression accounts for age, histology, and sample site. ***, $P < 0.001$; **, $P < 0.01$; *, $P < 0.05$.

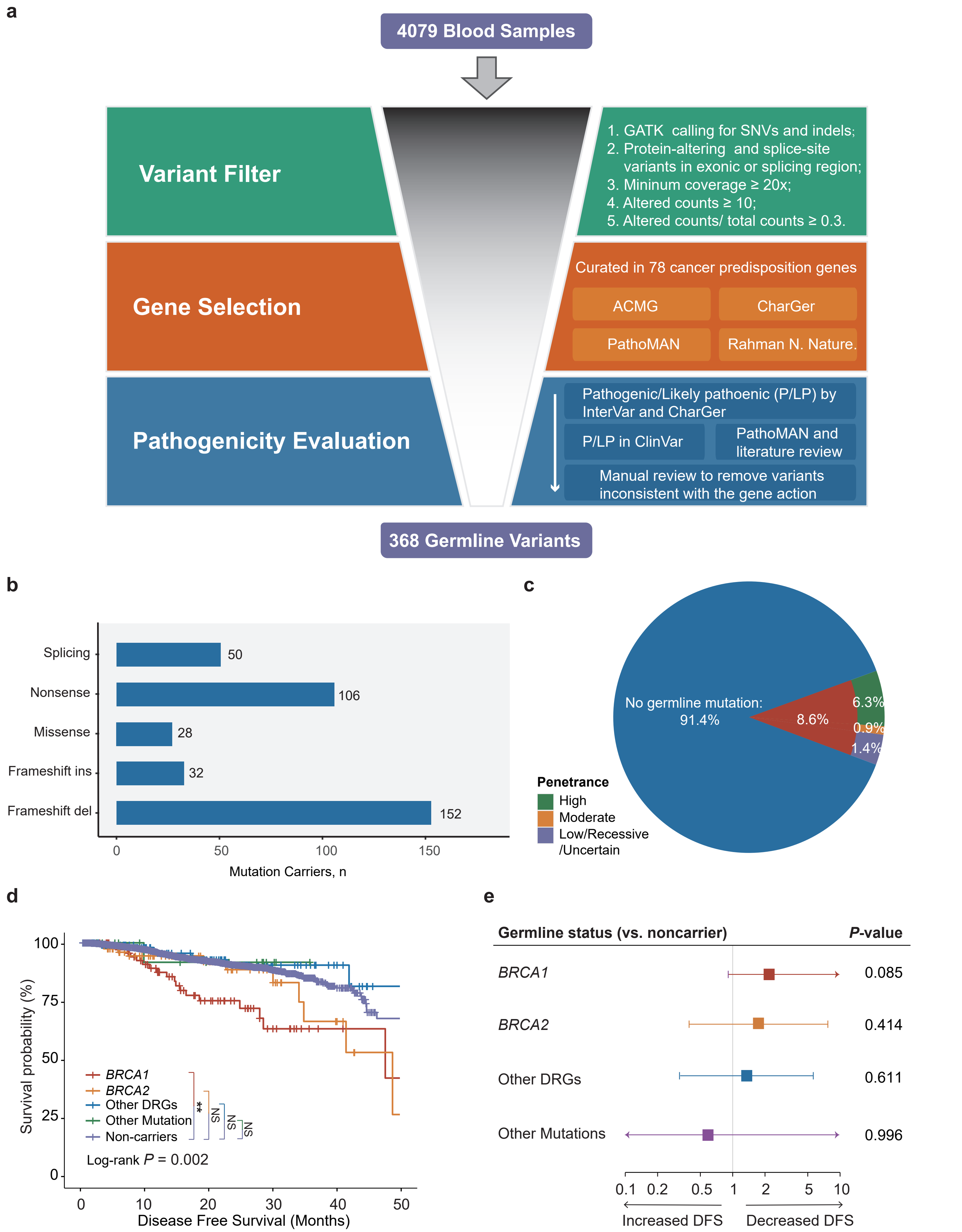

Supplementary Fig. S3. Workflow of germline variant calling in the FUSCC-BC cohort. a Flow diagram for calling germline mutations in the FUSCC-BC program. **b** Distribution of mutational types of pathogenic mutations. Abbreviations: del, deletion; ins, insertion. **c** Frequency and penetrance of germline mutations in breast cancer patients. **d** Kaplan-Meier curves of DFS in patients who have different germline mutation statuses. *BRCA1* versus non-carriers: ***P* = 0.0031, NS indicates $P > 0.05$. DRGs mean DNA repair genes. **e** A multivariate analysis of DFS based on germline status and other clinical characteristics showed a hazard ratio with 95% CIs. The adjustment for other clinical characteristics included age, family history of breast or ovarian cancer (FBOC), nuclear grade, tumor size, lymph node status, ER, PR, HER2 status, and treatment.

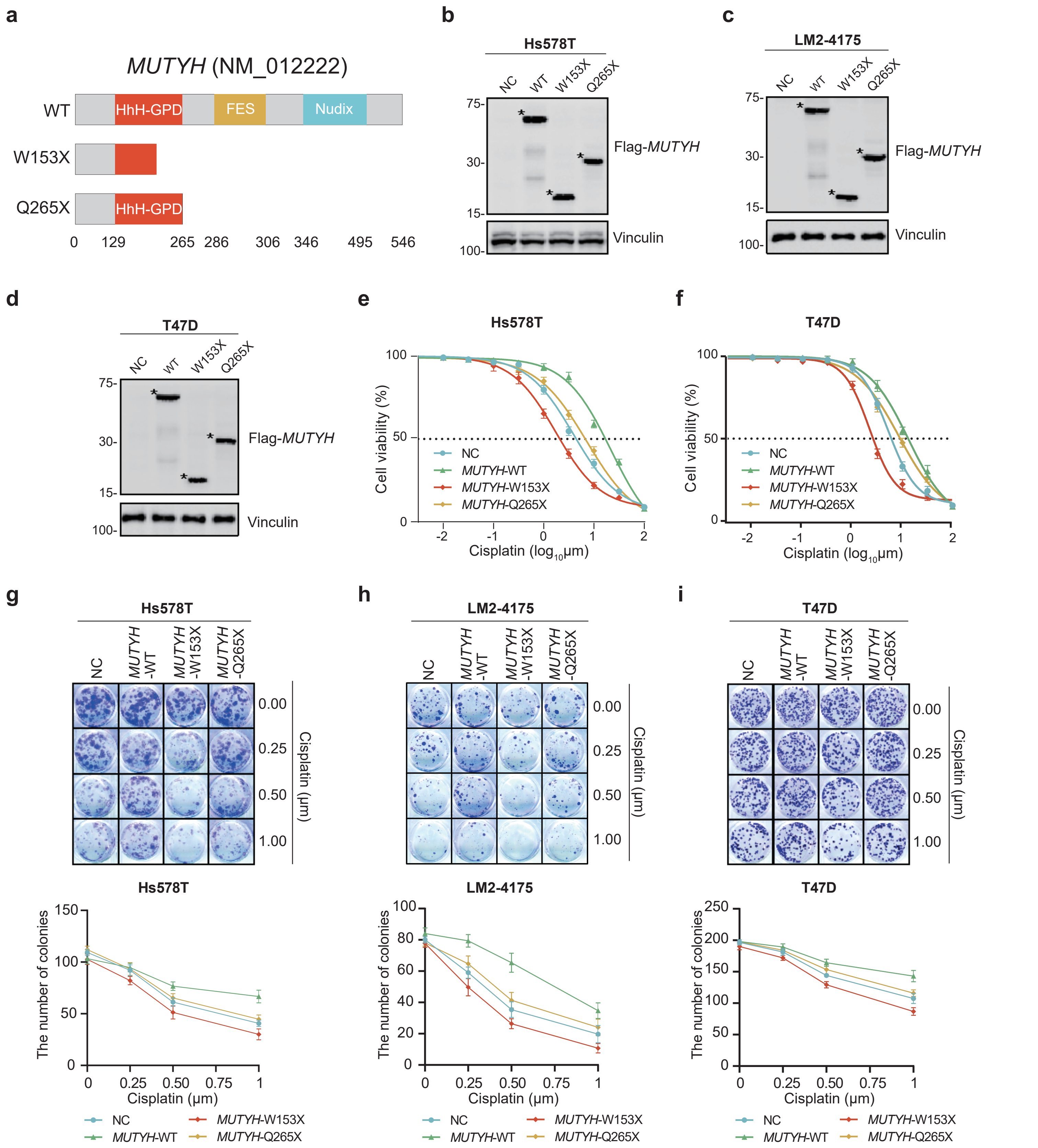

Supplementary Fig. S4. **Platinum sensitivity of *MUTYH* variants. a** Schematic of *MUTYH* WT and *MUTYH* pathogenic variants. **b-d** Hs578T, LM2-4175 and T47D cells transfected with empty vector, *MUTYH* WT, *MUTYH* W153X, and *MUTYH* Q265X. **e**,**f** Cell viability following treatment with cisplatin at the indicated concentration measured in Hs578T and T47D cells, which stably express empty vector, *MUTYH* WT, *MUTYH* W153X, and *MUTYH* Q265X. **g-i** After transfection with the empty vector, *MUTYH* WT, *MUTYH* W153X, and *MUTYH* Q265X, Hs578T, LM2-4175, and T47D cells were cultured with cisplatin at the indicated concentration for an additional 14 days and stained with crystal violet to determine colony formation capacity.

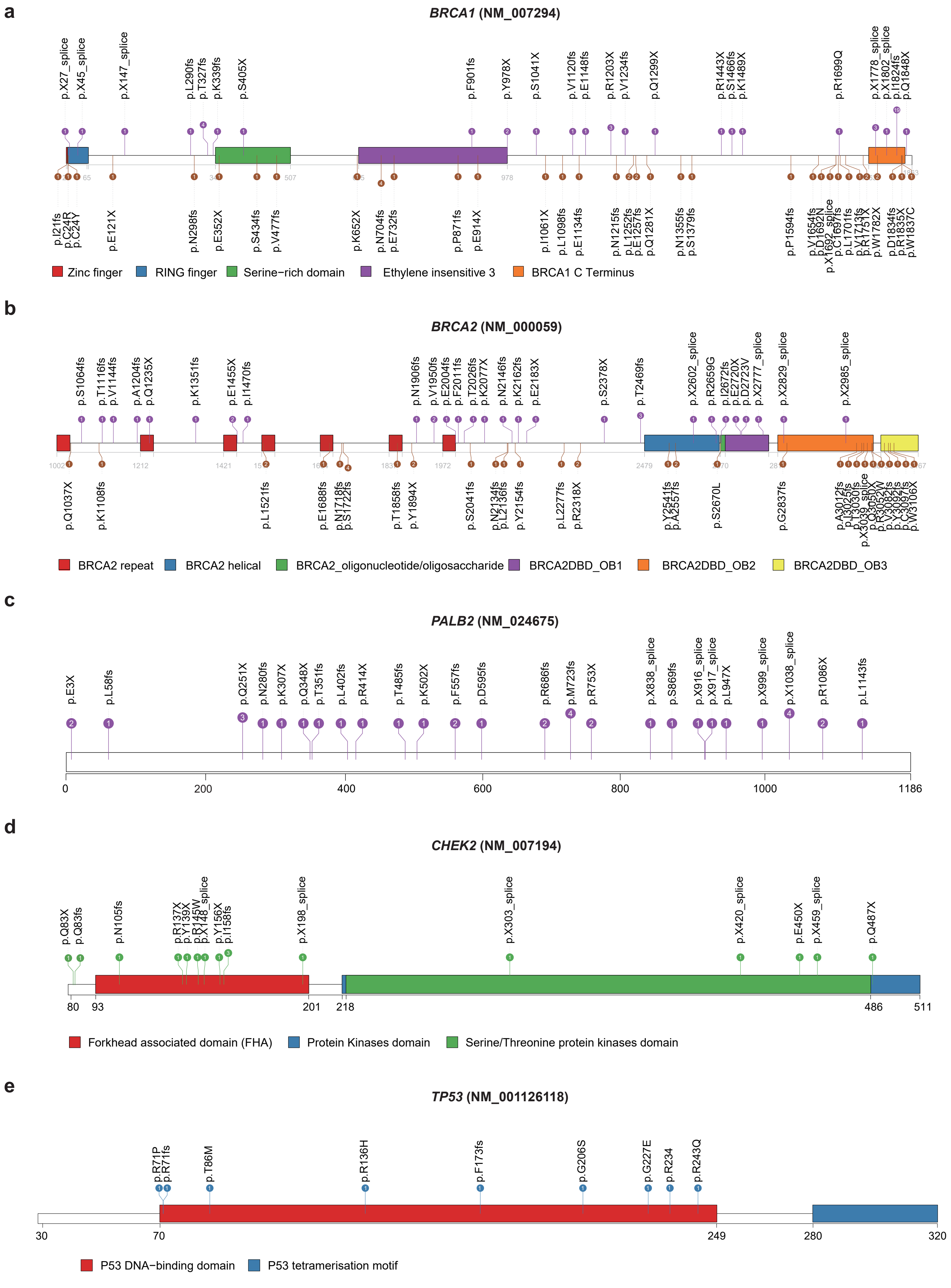

Supplementary Fig. S5. Pathogenic/likely pathogenic variants in genes associated with breast cancer risk. a-e Each variant in five BCDGs, including *BRCA1* (**a**), *BRCA2* (**b**), *PALB2* (**c**), *CHEK2* (**d**), and *TP53* (**e**), and protein domains is exhibited by lollipop plots. The values within the dots represent the mutation number of variants.

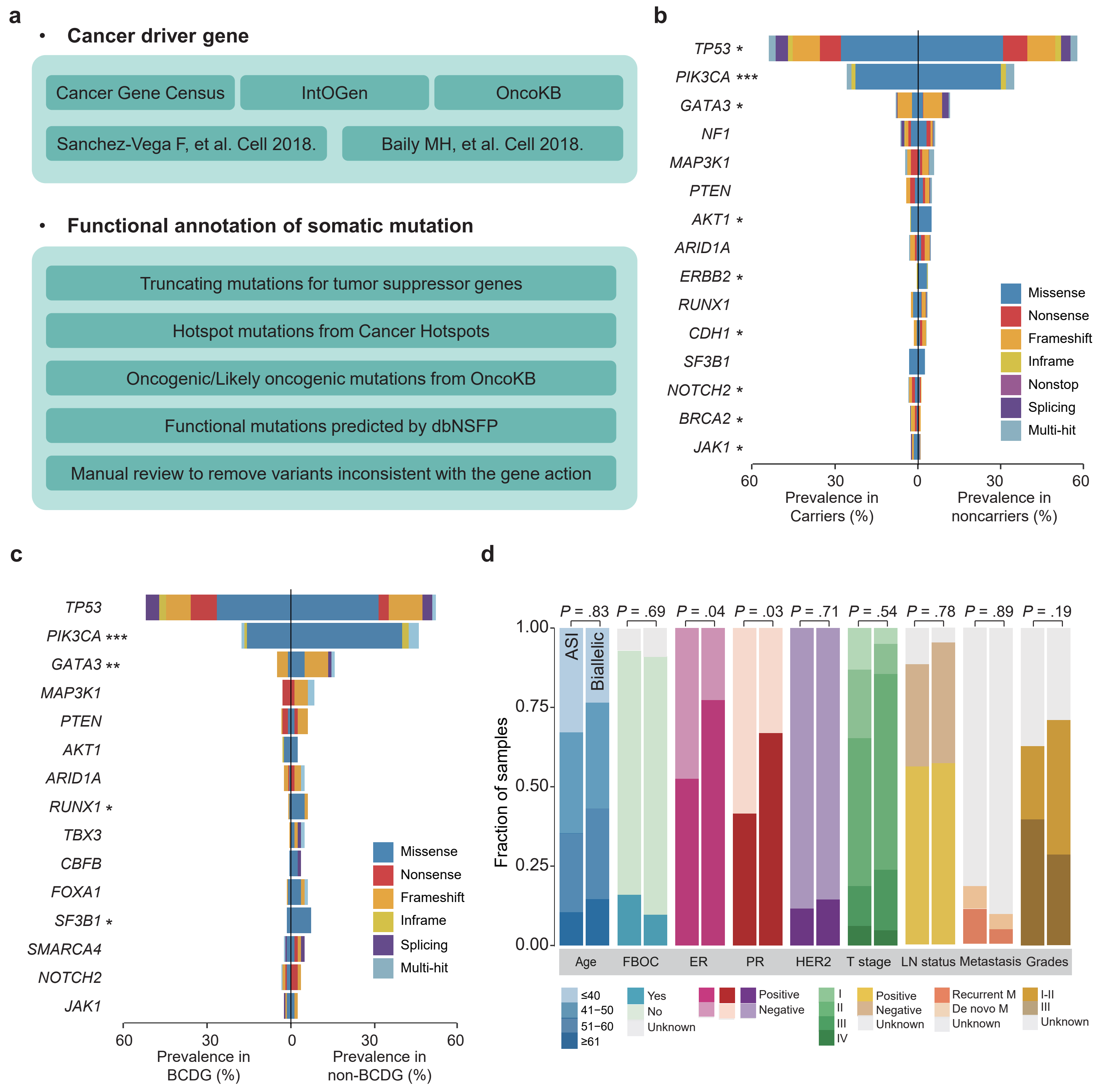

Supplementary Fig. S6. Flowchart of pathogenicity prioritization for somatic mutations, comparison of driver somatic mutations by germline status and comparisons of clinical features between ASI and biallelic mutation carriers. a Functional annotations of somatic mutations for cancer driver genes. **b,c** Comparison of driver somatic alterations between germline mutation carriers and noncarriers (**b**), the bar plots refer to the prevalence of somatic mutations in overall germline mutation carriers or non-carriers. Comparison of driver somatic alterations between germline BCDG carriers and non-BCDG carriers (**c**). Logistic regression accounts for histology and clinical subtypes. *, $P < 0.05$; **, $P < 0.01$; ***, $P < 0.001$. **d** Comparisons of clinical features between ASI and biallelic mutation carriers. P values were determined by Fisher's exact test.

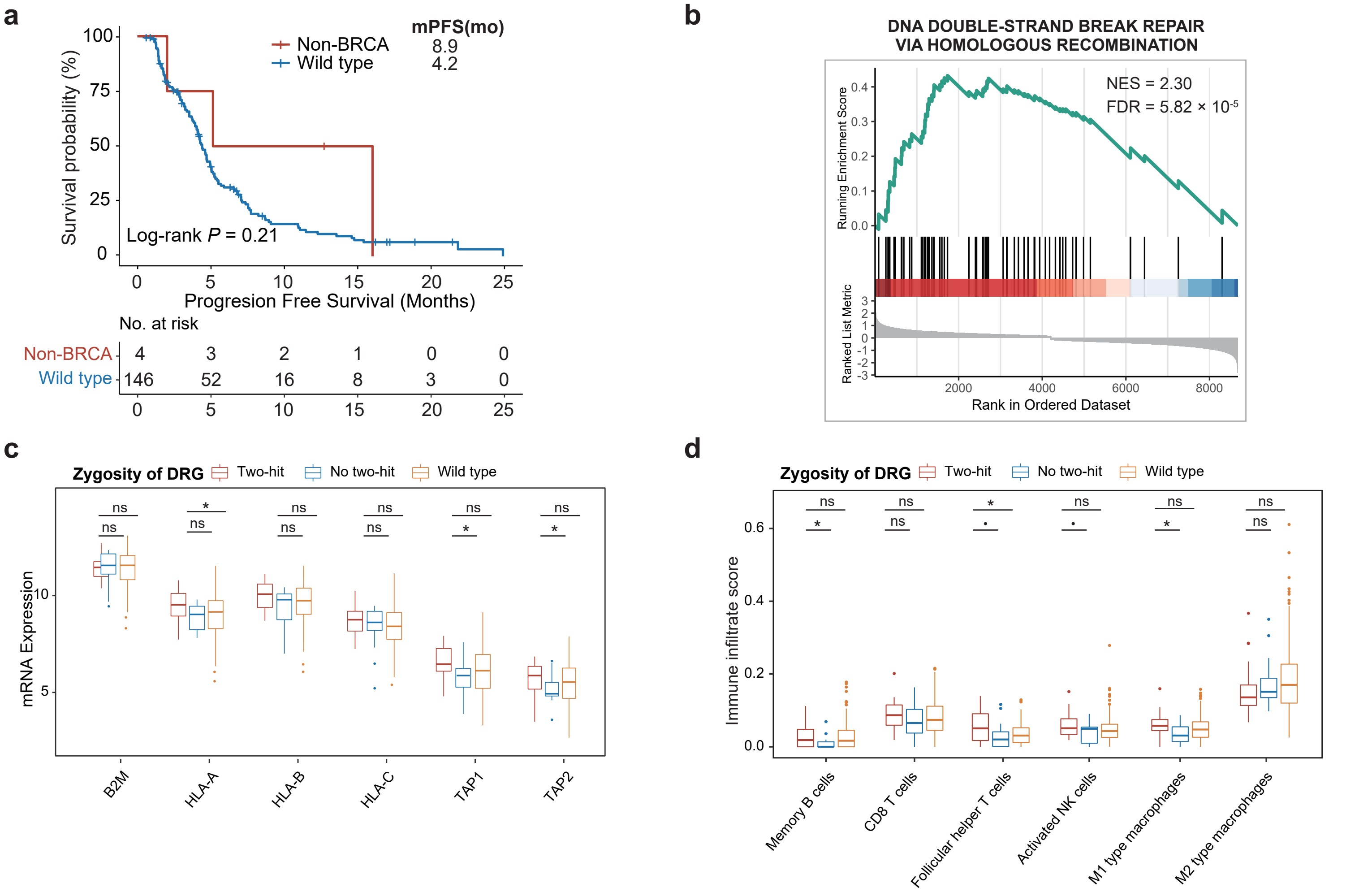

Supplementary Fig. S7. Extended analysis of BCDG mutation and two-hit inactivation. a Kaplan–Meier curves of PFS by germline mutation category in the platinum-treated advanced cohort of HER2-negative patients. non-BRCA: non-BRCA1/2 BCDGs, including *PALB2*, *CHEK2*, and *TP53*. **b** GSEA showing upregalated pathways of DNA repair in two-hit carriers by mRNA abundance. **c** Comparison of MHC class I molecular level among the groups of DNA repair gene (DRG) mutation carriers with two-hit inactivation, DRG carriers without two-hit inactivation, and noncarriers. **d** Comparison of the abundance of immune cell types estimated by CIBERSORT. •, $P < 0.1$; *, $P < 0.05$; ns, not significant.
